# Regulation of pneumococcal epigenetic and colony phases by multiple two-component regulatory systems

**DOI:** 10.1371/journal.ppat.1008417

**Published:** 2020-03-18

**Authors:** Juanjuan Wang, Jing-Wen Li, Jing Li, Yijia Huang, Shaomeng Wang, Jing-Ren Zhang

**Affiliations:** Center for Infectious Disease Research, School of Medicine, Tsinghua University, Beijing, China; Boston Children’s Hospital, UNITED STATES

## Abstract

*Streptococcus pneumoniae* is well known for phase variation between opaque (O) and transparent (T) colonies within clonal populations. While the O variant is specialized in invasive infection (with a thicker capsule and higher resistance to host clearance), the T counterpart possesses a relatively thinner capsule and thereby higher airway adherence and colonization. Our previous study found that phase variation is caused by reversible switches of the “opaque ON-or-OFF” methylomes or methylation patterns of pneumococcal genome, which is dominantly driven by the PsrA-catalyzed inversions of the DNA methyltransferase *hsdS* genes. This study revealed that switch frequency between the O and T variants is regulated by five transcriptional response regulators (*rr*) of the two-component systems (TCSs). The mutants of *rr06*, *rr08*, *rr09*, *rr11* and *rr14* produced significantly fewer O and more T colonies. Further mutagenesis revealed that RR06, RR08, RR09 and RR11 enrich the O variant by modulating the directions of the PsrA-catalyzed inversion reactions. In contrast, the impact of RR14 (RitR) on phase variation is independent of PsrA. Consistently, SMRT sequencing uncovered significantly diminished “opaque ON” methylome in the mutants of *rr06*, *rr08*, *rr09* and *rr11* but not that of *rr14*. Lastly, the phosphorylated form of RR11 was shown to activate the transcription of *comW* and two sugar utilization systems that are necessary for maintenance of the “opaque ON” genotype and phenotype. This work has thus uncovered multiple novel mechanisms that balance pneumococcal epigenetic status and physiology.

## Author summary

Human pathogen *Streptococcus pneumoniae* undergoes reversible switches or phase variation between opaque (O) and transparent (T) colonies within clonal populations. Phase variation is caused by reversible DNA methylation patterns or epigenetic status of pneumococcal genome, which is driven by the recombinase PsrA-catalyzed site-specific recombinations. This work has discovered that switch frequency between the O and T variants is regulated by five two-component transcriptional regulators. While RR06, RR08, RR09 and RR11 act on the PsrA-catalyzed inversion reactions, RR14 impacts phase variation in an inversion-independent manner. Further analysis indicated that the phosphorylated RR11 regulator enforces the O colony phase by activating the transcription of multiple pneumococcal gene loci that are necessary for the O colony genotype and phenotype. Because two-component systems represent a fundamental mechanism for bacterial sensing and responding to environmental conditions, this work has thus uncovered a new level of molecular sophistication and complexity in bacterial fine-tuning of its biological activities and behaviors by linking the environmental sensing systems to the epigenetic switching machinery.

## Introduction

*Streptococcus pneumoniae* (pneumococcus) is a commensal at human nasopharynx and also an important opportunistic pathogen that causes pneumonia, meningitis, septicemia, and otitis media [1]. This bacterium is well known for its strain-to-strain genetic variations, which are predominantly driven by natural transformation-mediated horizontal gene transfer. These variations are exemplified by acquisition of the exogenous genes conferring antimicrobial resistance, change of capsule types, and permutation of surface-exposed antigens. In the absence of foreign DNA, the pneumococcus is also capable of phase variation or reversible switch between the opaque (O) and transparent (T) colony variants in clonal populations on translucent agar plates [2, 3]. While the O colony variant is specialized in invasive infection (with a thicker capsule and higher resistance to host clearance), the transparent counterpart is characterized by a relatively thinner capsule, more cell wall teichoic acids, and thereby higher airway adherence [2–4]. In animal models, the O variant is more virulent in systemic infection while the T variants exhibit higher levels of nasopharyngeal colonization with relatively lower virulence [3, 4]. Recent studies have revealed that pneumococcal phase variation is epigenetically defined by reversible switches of the methylomes or genome methylation patterns [5, 6], which is controlled by invertase PsrA-catalyzed inversions of three homologous methyltransferase *hsdS* genes in the colony opacity determinant (*cod*) locus, also referred to as Spn556II locus [5, 7, 8].

The *cod* locus, a type-I restriction-modification (RM) system, consists of the *hsdR* (restriction endonuclease), *hsdM* (DNA methyltransferase), *psrA* (invertase), and three homologous *hsdS* (*hsdS*_*A*_, *hsdS*_*B*_ and *hsdS*_*C*_) genes [5]. PsrA catalyzes extensive inversions between *hsdS*_*A*_ and two downstream homologs (*hsdS*_*B*_ and *hsdS*_*C*_) [9, 10]. As a typical sequence recognition or S subunit of the type-I RM DNA methyltransferase (MTase) [11], *hsdS*_*A*_ encodes two target recognition domains (TRDs), each of which recognizes a half of the type-I RM methylation bipartite sequence [5]. Our recent study has shown that only *hsdS*_*A*_ is actively transcribed and produces a functional S subunit for the MTase, whereas *hsdS*_*B*_ and *hsdS*_*C*_ merely serve as templates for replacement of one or two TRD domains in the HsdS_A_ MTase by the PsrA-catalyzed inversions [5, 9]. The inversions generate six different *hsdS*_*A*_ alleles, each of which drives methylation of a unique DNA motif in the genome of *S*. *pneumoniae* [5, 7]. This site-specific recombination mechanism enables a single pneumococcal cell to generate a mixture of progeny cells each carrying one of the six *hsdS*_*A*_ alleles (A1-A6) and thereby producing six distinct methylomes [5]. Only the pneumococci carrying *hsdS*_*A1*_ form O colonies and those with the other five *hsdS*_*A*_ alleles produce T colonies [5, 6]. Therefore, the reversible switch between O and T colony phases is epigenetically driven by reversible “ON-or-OFF” alteration of the *hsdS*_*A1*_ genotype in the *cod* locus [5], although the biochemical basis of the colony opacity remains to be characterized.

The two-component signal transduction systems (TCSs) are widely found in bacteria to regulate a variety of cellular processes in response to changes in environmental conditions, such as chemotaxis, capsule production, balance of osmolarity, photosynthesis, sporulation and transformation [12]. A typical TCS is comprised of a histidine kinase (HK) as a sensor and a cognate response regulator (RR) as an effector. The histidine kinase auto-phosphorylates itself at the conserved histidine residue when it senses extracellular changes and the cognate response regulator is subsequently activated by accepting the phosphoryl group from histidine to its conserved aspartic acid [13]. The DNA-binding output domain of activated regulator typically interacts with the promoter(s) of the target genes and activates/represses their transcription. *S*. *pneumoniae* has 13 complete TCSs and an orphan regulator (RR14, RitR) [14–16]. Previous studies have shown that most of the TCSs are necessary for pneumococcal pathogenicity in animal models [15, 17], but the precise functions for most of the TCSs are largely unknown.

TCS05 (CiaR/H) and TCS12 (ComE/D) represent the best-characterized pneumococcal TCSs. TCS05 inhibits the competence state [18–21], and promotes pneumococcal resistance to certain antibiotics and other stressful conditions [20, 22–24]. TCS12 activates the genes associated with the competence state and natural transformation [25–27]. TCS02 (WalR/K), the only TCS essential for pneumococcal viability, is associated with cell wall peptidoglycan metabolism [28, 29]; TCS03 (LiaR/S) senses cell wall damage and inhibits autolysis [30], TCS04 (PnpR/S) regulates uptake of inorganic phosphate [31, 32]; TCS06 (CbpR/S) activates expression of choline-binding protein A or PspC [33, 34]; TCS08 is involved in the cellobiose uptake [35], production of pilus-1 [16] and surface protein PavB [16]; TCS13 (BlpR/H) regulates the production of bacteriocins (pneumocins) [36–38]. The orphan response regulator RR14 represses the transcription of the pneumococcal iron uptake (Piu) and other nutrient uptake loci in response to oxidative stress [39, 40]. The cellular functions of the remaining five TCSs (1, 7, 9, 10 and 11) are poorly characterized. TCS09 appears to regulate transcription of genes involved in sugar transport, competence and pilus-1 production in a strain-dependent manner [41, 42], whereas the protein structure of RR11 has been described without a known function [43]. In this study, we systematically determined the impact of 13 pneumococcal response regulators (except for the essential RR02) on phase variation in colony opacity. Five of those response regulators were found to enhance the O colony phase, mostly through modulation of PsrA-catalyzed inversions in the *cod* locus.

## Results

### Five response regulators modulate pneumococcal colony phases

**Fig 1:**
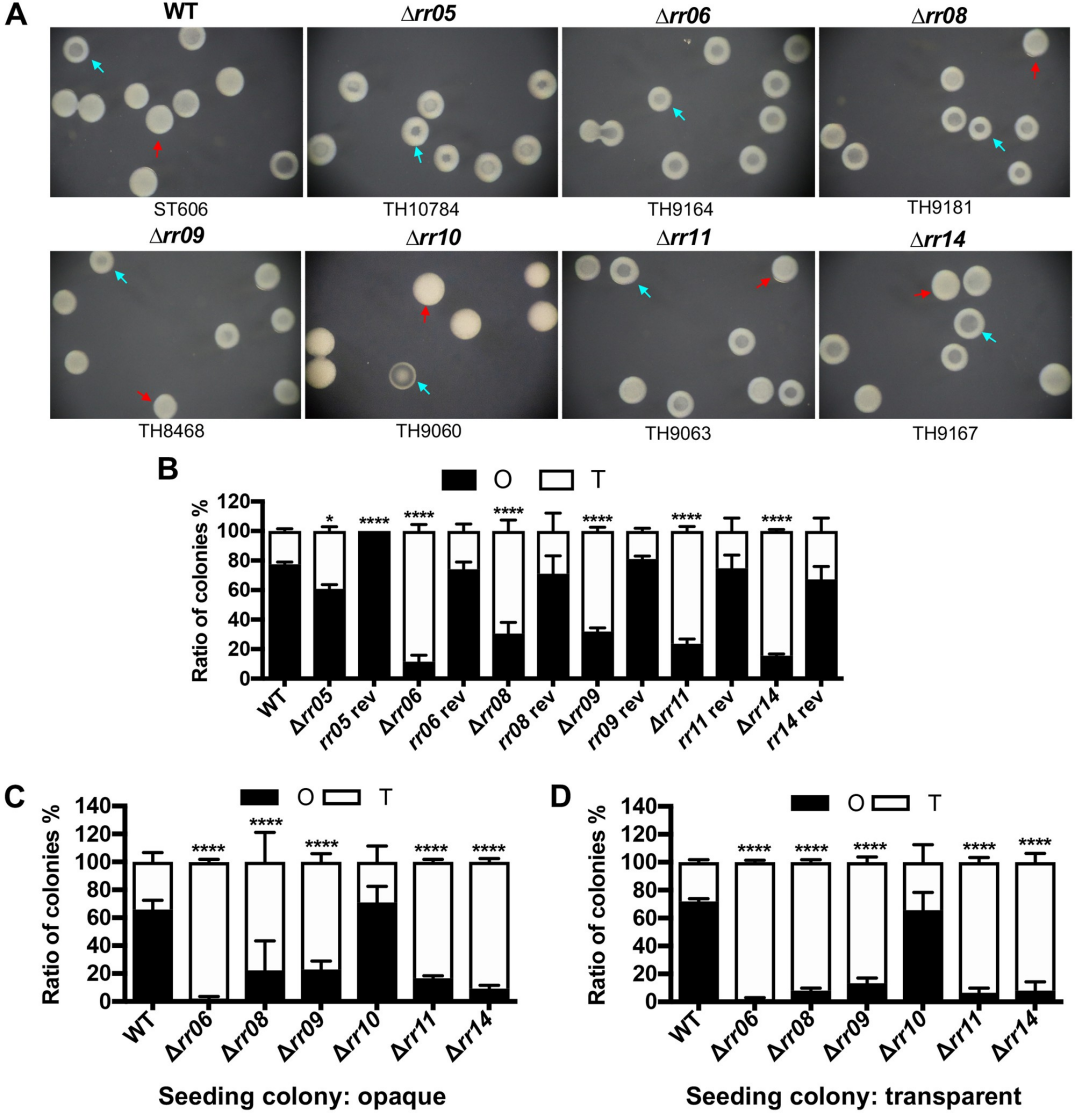
Colony phenotypes of the *rr* mutants. **A**. Representative colonies formed by selected *rr* mutants. ST606 (WT) and isogenic *rr* mutants were separately spread on catalase-TSA plates, incubated for 17 hrs before the colonies were photographed under a dissection microscope. Genotype (top) and identification (bottom) of each strain are marked. Representative colonies are indicated by red (opaque) and light blue (transparent) arrowheads, respectively. **B**. Ratio between opaque (O) and transparent (T) colonies of selected *rr* mutants and their revertants. O and T colonies on each plate were prepared as in Fig 1A to quantify O and T colonies formed by ST606 (WT), isogenic *rr* mutants and their revertants. The mean ± SEM of 3 values (from 3 plates) for the O (filled) and T (open) colonies of each strain is presented in a single bar. **C**. Ratio between O and T colonies derived from single O seeding colonies of the six selected *rr* mutants. Three well-separated O colonies were spread on three catalase-TSA plates to assess the relative ratio between O and T colonies for each strain as described in Fig 1B. **D**. Same as Fig 1C except for using T colonies as the seeding bacteria.

To determine potential impact of pneumococcal TCSs on phase variation in colony opacity, we generated unmarked deletion mutants of 13 response regulator (*rr*) genes in strain ST606, a streptomycin-resistant *rpsL1* derivative of type-19F strain ST556, except for the essential gene *rr02* [14, 44]. The rationale was that disruption of the *rr* gene in bacterial TCS is more likely to yield a functional phenotype [45]. The colony phenotype or ratio between O and T colonies in a single clonally derived population was characterized for each mutant on the catalase-TSA plates. Similar to the parental strain ST606, seven of the 13 *rr* mutants generated populations that were dominated by O colonies (*rr01*, *rr03*, *rr04*, *rr07*, *rr10*, *rr12* and *rr13*) (Table 1). As an example, the *rr10* mutant produced 75.2% O and 24.8% T colonies, which is comparable with the parental strain (79.8% O and 20.2% T). In contrast, deleting the six other regulators (*rr05*, *rr06*, *rr08*, *rr09*, *rr11* and *rr14*) led to significantly reduced proportion of O colonies as compared with the parental strain (Fig 1A). The % O values of the *rr05*, *rr06*, *rr08*, *rr09*, *rr11* and *rr14* mutants are 60.9, 9.0, 14.5, 17.0, 22.2 and 27.8, respectively (Table 1). It should be noted that the transparent colonies of the *rr05* mutant were morphologically different from the parental counterpart. ST606 produced transparent colonies with a large translucent center and a thin halo around it, but the *rr05* counterparts showed a relatively smaller transparent center and a thicker ring (Fig 1A).

**Table 1:** The ratios of T and O colonies formed by the *rr* mutants.

Strain	Genotype	Number of colonies^1^		%^2^ Transparent	%^3^ Opaque	T/O ratio	*P* value
		T	O				
ST606	ST556 *rpsL1*	80	310	20.2	79.8	1:3.9	NR^4^
TH9048	Δ*rr01*	106	516	16.9	83.1	1:4.9	0.5849
TH7009	Δ*rr03*	166	459	26.5	73.5	1:2.8	0.2431
TH9054	Δ*rr04*	33	145	18.3	81.7	1:4.4	0.7185
**TH10784**	**Δ*rr05***	**219**	**342**	**39.1**	**60.9**	**1:1.6**	**0.0032**
**TH9164**	**Δ*rr06***	**391**	**36**	**91.0**	**9.0**	**1:0.1**	**<0.0001**
TH9057	Δ*rr07*	20	118	14.8	85.2	1:5.9	0.3521
**TH9181**	**Δ*rr08***	**243**	**41**	**85.5**	**14.5**	**1:0.2**	**<0.0001**
**TH8468**	**Δ*rr09***	**325**	**71**	**83.0**	**17.0**	**1:0.2**	**<0.0001**
TH9060	Δ*rr10*	107	322	24.8	75.2	1:3.0	0.3972
**TH9063**	**Δ*rr11***	**108**	**32**	**77.8**	**22.2**	**1:0.3**	**<0.0001**
TH9259	Δ*rr12*	68	141	32.4	67.6	1:2.1	0.0531
TH9066	Δ*rr13*	69	172	29.0	71.0	1:2.5	0.139
**TH9167**	**Δ*rr14***	**286**	**111**	**72.2**	**27.8**	**1:0.4**	**<0.0001**

^1^Each number represents an average of the colonies from three plates in a representative experiment.

^2, 3^Average transparent and opaque colony ratio were calculated by the average of followed values from three plates: number of each form of colony divided by the total colony number *100%.

^4^NR: not relevant because it was used as a base value for comparison with the mutants.

To verify the impact of these six regulators on pneumococcal colony opacity, we constructed genetic revertants of these mutants by replacing each deletion with the corresponding wild type gene in its native locus of the genome. The revertants of the *rr06*, *rr08*, *rr09*, *rr11* and *rr14* mutants displayed a comparable fraction of O colonies as the parental strain (Fig 1B; S1 Fig; S1 Table). However, the *rr05* revertant strain produced 100.0% O colonies. Whole genome sequencing of the *rr05* mutant and its revertant revealed a single nucleotide mutation from adenine to cytosine at the 539^th^ position in the coding region of MYY890, which encodes the substrate-binding protein in the phosphate uptake system [31]. This nonsynonymous mutation led to a substitution of the 180^th^ glutamine (CAG) with proline (CCG) of this protein. It is worth of mentioning that other pneumococcal strains lacking *rr05* also tended to carry spontaneous mutations in our collection. These findings strongly suggested that deleting *rr05* somehow renders the genome more prone to spontaneous mutations, which makes it difficult to properly interpret the colony morphology data. The TCS05 was thus excluded from further investigation. These results demonstrated that RR06, RR08, RR09, RR11 and RR14 regulate pneumococcal colony opacity.

We next determined the impact of the five regulators on reversibility between O and T colonies by re-streaking individual O and T colonies of each mutant on catalase-TSA plates. For each of the five mutants, the progeny colonies derived from both the O and T colony seeds showed a comparable ratio between the O and T phenotypes. As an example, the populations derived from the O (Fig 1C) and T (Fig 1D) seeding colonies of the *rr06* mutant showed the most dramatic but similar reduction in the fraction of O colonies (1.9% for O seed, 1.6% for T seed). This finding is consistent with the result from the initial screening of the *rr06* mutant (Fig 1B). As a negative control, progeny colonies of the *rr10* mutant did not show significant alteration in colony phenotype, regardless the original phenotypes of the seeding colonies (Fig 1C and 1D). This result indicated that the *rr06*, *rr08*, *rr09*, *rr11* and *rr14* mutants still retained the capability of phase switching.

### Colony phases are regulated by the PsrA-dependent and -independent mechanisms

**Fig 2:**
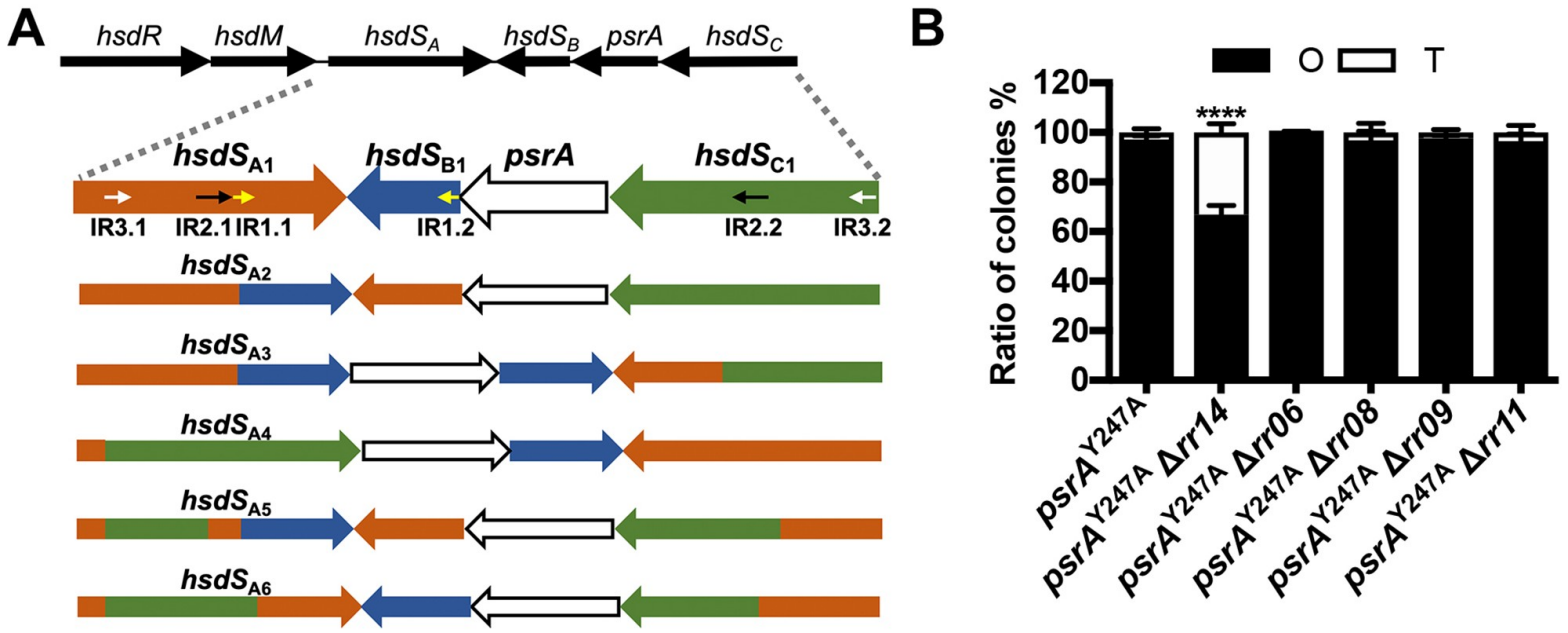
Characteristics of the colonies produced by the *psrA*^Y247A^-*rr* double mutants. **A**. Schematic illustration of the gene organization in the *cod* locus. The genes encoding the restriction enzyme (*hsdR*), DNA methyltransferase (*hsdM*), sequence recognition proteins (*hsdS*_*A*_, *hsdS*_*B*_ and *hsdS*_*C*_) and invertase (*psrA*) are depicted as thin arrows at the top. Three pairs of inverted repeats (IR1, IR2 and IR3) flanking the invertible regions that mediate DNA inversions are indicated as small arrowheads in the first row of the lower panel which depicts the six major *hsdS*_*A*_ allelic configurations generated by PsrA-catalyzed inversions. **B**. Ratio between O and T colonies produced by the five selected *rr* mutants that were generated in the *psrA*^Y247A^ background. The colonies were prepared and processed as in Fig 1B.

Our previous study has found that the reversible switch between O and T colony phenotypes is controlled by the PsrA-catalyzed inversions of the three invertible regions in the *cod* locus [5, 9] (Fig 2A). Therefore, enzymatic inactive *psrA*^Y247A^ mutant (with a point mutation in the catalytic residue tyrosine 247) is locked in either O or T colony phase [9]. To determine whether the impact of RR06, RR08, RR09, RR11 and RR14 on phase variation requires PsrA-mediated inversions, we constructed deletion mutant in each of these genes in the O-locked *psrA*^Y247A^ strain, which was previously shown to produce only the O colonies [5] (Fig 2B; S2 Fig). Additional deletion of *rr14* in this inversion-deficient mutant still resulted in significant changes in colony phenotype. To a less extent, the Δ*rr14-psrA*^Y247A^ mutant formed 66.9% O colonies. This result suggested that RR14 (RitR) regulates pneumococcal colony opacity in a PsrA (*hsdS* inversion)-independent manner. However, deleting *rr06*, *rr08*, *rr09* or *rr11* in the *psrA*^Y247A^ background did not yield any obvious impact on the phenotype of the strain. Virtually all of the colonies formed by these four double mutants showed the O phenotype (Fig 2B; S2 Fig). This result indicated that regulatory impact of RR06, RR08, RR09 and RR11 on phase variation depends on the PsrA-mediated *hsdS* inversions.

### The PsrA-dependent regulators modulate pneumococcal methylome

Because only the methylome specified by the HsdS_A1_ MTase is required for the formation of O colonies [5], the abovementioned changes of the *rr* mutants in colony phenotypes strongly suggested that these response regulators regulate pneumococcal methylome through PsrA-driven inversions. We thus compared the methylomes of strain ST606 and 13 isogenic *rr* mutants by single molecule real-time (SMRT) sequencing. This trial detected N6-methyladenine (6-mA) in virtually all 664 loci of the Spn556I recognition motif (5’-TCTAG^m6^A-3’, type II RM) in ST606 and its 13 derivatives (S2 Table), and thereby demonstrated that our sequencing setup was adequate for genome-wide detection of all 6-mA methylated sequences.

**Fig 3:**
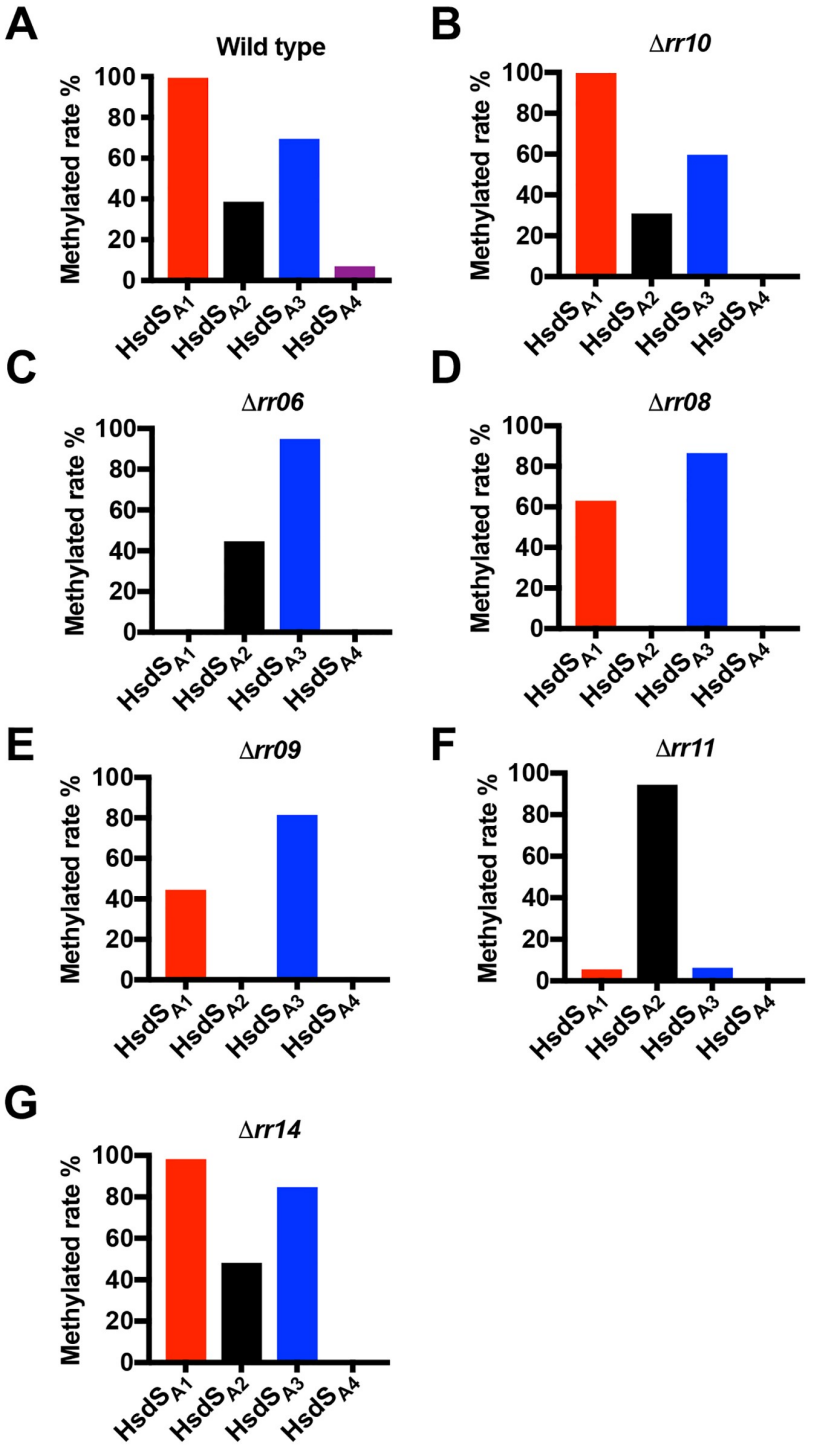
Relative methylation rate of the DNA motifs recognized by four HsdS_A_ allelic variants in the *rr* mutants. Relative methylation rate of each DNA motif recognized by HsdS_A1_ (5’-CRA^m6^AN_8_CTT-3’/3’-GYTTN_8_G^m6^AA-5’, 2,058 loci), HsdS_A2_ (5’-CRA ^m6^AN_9_TTC-3’/3’-GYTTN_9_^m6^AAG-5’, 2,054 loci), HsdS_A3_ (5’-CRA^m6^AN_8_CTG-3’/3’-GYTTN_8_G ^m6^AC-5’, 1,468 loci) and HsdS_A4_ (5’-C ^m6^ACN_7_CTG-3’/3’-GTGN_7_G ^m6^AC-5’, 888 loci) was calculated in each strain by dividing the number of methylated chromosomal loci for each motif with the total loci of the motif in the ST556 genome. Only the values for the parental strain ST606 (**A**) and isogenic mutants of *rr10* (**B**), *rr06* (**C**), *rr08* (**D**), *rr09* (**E**), *rr11* (**F**) and *rr14* (**G**) are presented. The results for the other *rr* mutants are described in Table 2.

Consistent with frequent *hsdS* inversions in the *cod* locus [5, 7], SMRT sequencing revealed 6-mA methylation in the motifs recognized by four of the six *hsdS*_A_ allelic variants (HsdS_A1_, HsdS_A2_, HsdS_A3_ and HsdS_A4_) of the *cod* locus in ST606 (Fig 3A; Table 2). Virtually all 2,058 loci of the HsdS_A1_ motif in the genome were methylated (99.4%) in ST606, but there were much lower methylation percentages for the other motifs (HsdS_A2_−38.7%, HsdS_A3_−69.5% and HsdS_A4_−7.0%); no 6-mA methylation was detected for any loci of the HsdS_A5_ and HsdS_A6_ motifs in ST606 or its 13 derivatives. This observation is consistent with the previous finding that single clonal population of ST606 is predominantly made up by the cells possessing the *hsdS*_A1_, *hsdS*_A2_ or *hsdS*_A3_ allele [5]. As exemplified with the *rr10* mutant in Fig 3B, nine of the 13 *rr* mutants (*rr01*, *rr03*, *rr04*, *rr05*, *rr07*, *rr10*, *rr12*, *rr13* and *rr14*) exhibited a similar methylome as the parental strain (Table 2, S2 and S3 Tables).

**Table 2:** Methylation sequences specified by the *cod* MTases^1^.

Genotype	HsdS_A1_			HsdS_A2_			HsdS_A3_			HsdS_A4_		
	5’-CRA^m6^AN_8_CTT-3’ 3’-GYTTN_8_G^m6^AA-5’			5’-CRA^m6^AN_9_TTC-3’ 3’-GYTTN_9_^m6^AAG-5’			5’-CRA^m6^AN_8_CTG-3’ 3’-GYTTN_8_G^m6^AC-5’			5’-C^m6^ACN_7_CTG-3’ 3’-GTGN_7_G^m6^AC-5’		
	# in genome^2^	# detected^3^	% detected^4^	# in genome	# detected	% detected	# in genome	# detected	% detected	# in genome	# detected	% detected
Wild type	2058	2045	99.4	2054	794	38.7	1468	1020	69.5	888	62	7.0
Δ*rr01*	2058	2036	98.9	2054	1241	60.4	1468	1071	73.0	888	745	83.9
Δ*rr03*	2058	2050	99.6	2054	467	22.7	1468	1462	99.6	888	0	0
Δ*rr04*	2058	2051	99.7	2054	343	16.7	1468	984	67.0	888	0	0
**Δ*rr05***	**2058**	**2056**	**99.9**	**2054**	**610**	**29.7**	**1468**	**1047**	**71.3**	**888**	**0**	**0**
**Δ*rr06***	**2058**	**0**	**0**	**2054**	**919**	**44.7**	**1468**	**1393**	**94.9**	**888**	**0**	**0**
Δ*rr07*	2058	2039	99.1	2054	164	8.0	1468	372	25.3	888	0	0
**Δ*rr08***	**2058**	**1299**	**63.1**	**2054**	**0**	**0**	**1468**	**1271**	**86.6**	**888**	**0**	**0**
**Δ*rr09***	**2058**	**916**	**44.5**	**2054**	**0**	**0**	**1468**	**1196**	**81.5**	**888**	**0**	**0**
Δ*rr10*	2058	2054	99.8	2054	634	30.9	1468	877	59.7	888	0	0
**Δ*rr11***	**2058**	**114**	**5.5**	**2054**	**1941**	**94.5**	**1468**	**93**	**6.3**	**888**	**0**	**0**
Δ*rr12*	2058	1975	96.0	2054	0	0	1468	401	27.3	888	0	0
Δ*rr13*	2058	2056	99.9	2054	796	38.8	1468	1120	76.3	888	0	0
**Δ*rr14***	**2058**	**2022**	**98.3**	**2054**	**989**	**48.1**	**1468**	**1243**	**84.7**	**888**	**0**	**0**

^1^The accumulative number of all methylated loci in each strain exceeded 100% because a base was considered as being methylated once more than 30% of all the reads at the position passed the cutoff value in the PacBio platform.

^2^Total number of loci in both DNA strands in the genome of ST556 (accession CP003357.2).

^3^Total loci detected by the SMRT sequencing.

^4^Percentage of the detected motifs was calculated as follows: total loci detected/total loci in the genome.

N = any nucleotide; R = A or G; Y = T or C.

In agreement with significant decrease in the fraction of O colonies in the *rr06*, *rr08*, *rr09* and *rr11* mutants, these strains showed dramatic alteration in the methylome specified by the “opaque ON” HsdS_A1_ MTase. The *rr06* mutant showed the most striking overhaul in the methylome (Fig 3C). Compared with 99.4% methylation rate of the HsdS_A1_ motif in ST606 (Fig 3A), none of these sequences were detected as a methylated form in this mutant. The complete loss of methylated HsdS_A1_ motif loci in this strain was accompanied with increase in methylation rates of the HsdS_A2_ (2,054 copies) and HsdS_A3_ (1,468 copies) motifs. Identification of 6-mA methylation in the HsdS_A2_ and HsdS_A3_ motifs demonstrated that the HsdM subunit of the *cod* MTase was functional in the *rr06* mutant. The complete loss of HsdS_A1_ motif methylation in the *rr06* mutant was consistent with dramatic reduction in the fraction of the O colonies in this strain (Fig 1; Table 1). Similar to their common characteristics in the colony phenotype (Fig 1; Table 1), the mutants of *rr08* and *rr09* showed a similar pattern in methylome. Methylation of the HsdS_A1_ motif loci fell to 63.1% and 44.5% in the *rr08* (Fig 3D) and *rr09* (Fig 3E) mutants, respectively. Both mutants also displayed a moderate increase in methylation of the HsdS_A3_ motif loci. Interestingly, none of the 2,054 HsdS_A2_ motif copies were methylated in either *rr08* or *rr09* (Table 2). In sharp contrast, nearly all of the HsdS_A2_ motif loci were methylated in the *rr11* mutant (94.5%) (Fig 3F). The methylation rates of the other HsdS_A_ motifs were extremely low (5.5% for HsdS_A1_ and 6.3% for HsdS_A3_) or undetectable (HsdS_A4-6_) in this strain. The methylation level of various HsdS_A_ motifs in the *rr11* mutant is consistent with its colony phenotype (Fig 1). In summary, the SMRT sequencing confirmed the observation with the *psrA*^*Y247A*^-*rr* double mutants that RR06, RR08, RR09 and RR11 regulate pneumococcal methylome through the PsrA-driven inversions.

In contrary to its T colony-dominant phenotype, the methylome of the *rr14* mutant was virtually identical to that of the parental strain (Table 2; S2 and S3 Tables). In particular, virtually all 2,058 copies of the HsdS_A1_ motif were methylated in the *rr14* mutant (Fig 3G). This result is consistent with their lack of association with PsrA-catalyzed inversions (Fig 2), and further validated our conclusion that RR14 impacts pneumococcal colony morphology through a non-epigenetic mechanism. DNA methylation detection also revealed certain interesting features associated with the Spn556III locus, the other functional type-I RM system in ST556 and many other pneumococcal strains [7, 46, 47]. A recent study reports that the two *hsdS* genes in this locus undergo excision and reintegration recombinations in other pneumococcal strains [47]. In addition to a previously identified motif (5’-GAT^m6^AN_7_TCA-3’) for this system [46], the parental (5’-GG^m6^AN_7_TGA-3’) and Δ*rr13* (5’-GG^m6^AN_7_TCA-3’) strains each showed a new type-I RM MTase recognition sequence (S3 Table). Based on the published specificities of the HsdS variants for this system [47], it is apparent that these sequences were methylated by two new HsdS alleles generated by DNA excisions in the Spn556III locus. We designated these HsdS alleles as the HsdS_1_ (published), HsdS_2_ (new in ST606) and HsdS_3_ (new in Δ*rr13*) (S3 Table). Complete shift of the methylation activity from the HsdS_1_ MTase to HsdS_3_ MTase in Δ*rr13* was caused by the formation of a hybrid *hsdS*_*3*_ allele in the Spn556III locus as reflected in the SMRT sequencing data. This observation suggested that RR13 influences the *hsdS* recombinations in the Spn556III locus. However, our preliminary mutagenesis trial did not reveal obvious connection between the Spn556III locus and colony phases. The mutants with unmarked deletion of the entire Spn556III locus displayed a similar methylation rate of the *cod* HsdS_A1_ motif to the parental strain.

### The PsrA-dependent regulators modulate the colony phases in multiple pneumococcal strains

**Fig 4:**
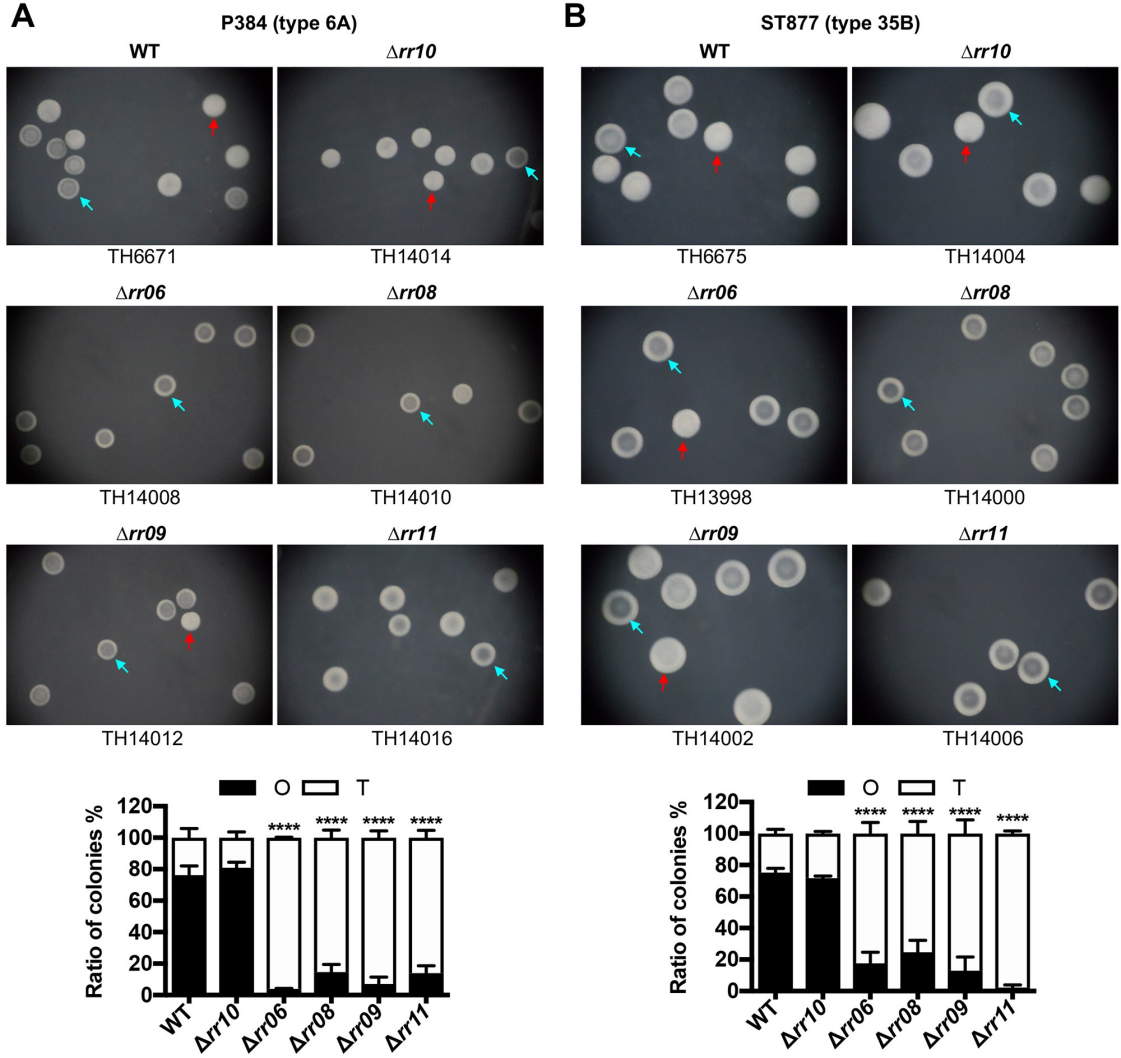
The colony characteristics of the *rr06*, *rr08*, *rr09* and *rr11* mutants in the P384 and ST877 strain backgrounds. **A**. Representative colonies formed by P384 (6A) and its isogenic *rr* mutants. The colonies were generated, photographed and marked as in Fig 1A. The ratio between the O and T colonies (bottom) is displayed as in Fig 1B except for different strains. **B**. Representative colonies produced by ST877 (35B) and its isogenic *rr* mutants. Same as in (A) with the exception of different strains.

Based on the significant impact of RR06, RR08, RR09 and RR11 on the directions of phase variation in the ST556 background, we tested the role of these four regulators in P384 (serotype 6A) and ST877 (serotype 35B), two strains that exhibited typical *hsdS*_*A1*_-dependent phase variation in colony opacity [5]. In agreement with our previous study [5], strain P384 produced 76.2% O colonies on catalase-TSA plates. However, deleting *rr06*, *rr08*, *rr09* or *rr11* led to dramatic reduction in the fraction of O colonies within clonal populations (Fig 4A). The mutants of *rr06*, *rr08*, *rr09* and *rr11* produced 3.9%, 14.6%, 7.1% and 13.9% O colonies, respectively. As a negative control, the *rr10* mutant retained a similar percentage of O colonies (80.9%) as the parental strain. In a similar manner, genetic deletions of *rr06*, *rr08*, *rr09* or *rr11* in strain ST877 also resulted in remarkable loss of O colonies (Fig 4B). As compared with the 75.3% O colonies produced by the parental strain, the mutants of *rr06*, *rr08*, *rr09* and *rr11* only formed 17.7%, 24.6%, 12.9% and 2.3% O colonies, respectively. In contrast, the isogenic *rr10* mutant of ST877 produced a similar level of O colonies (71.7%) as the parental strain. Together, these results demonstrated that RR06, RR08, RR09 and RR11 are required for the O phenotype in multiple pneumococcal strains.

### Phosphorylated form of RR11 drives pneumococci toward the O phase

**Fig 5:**
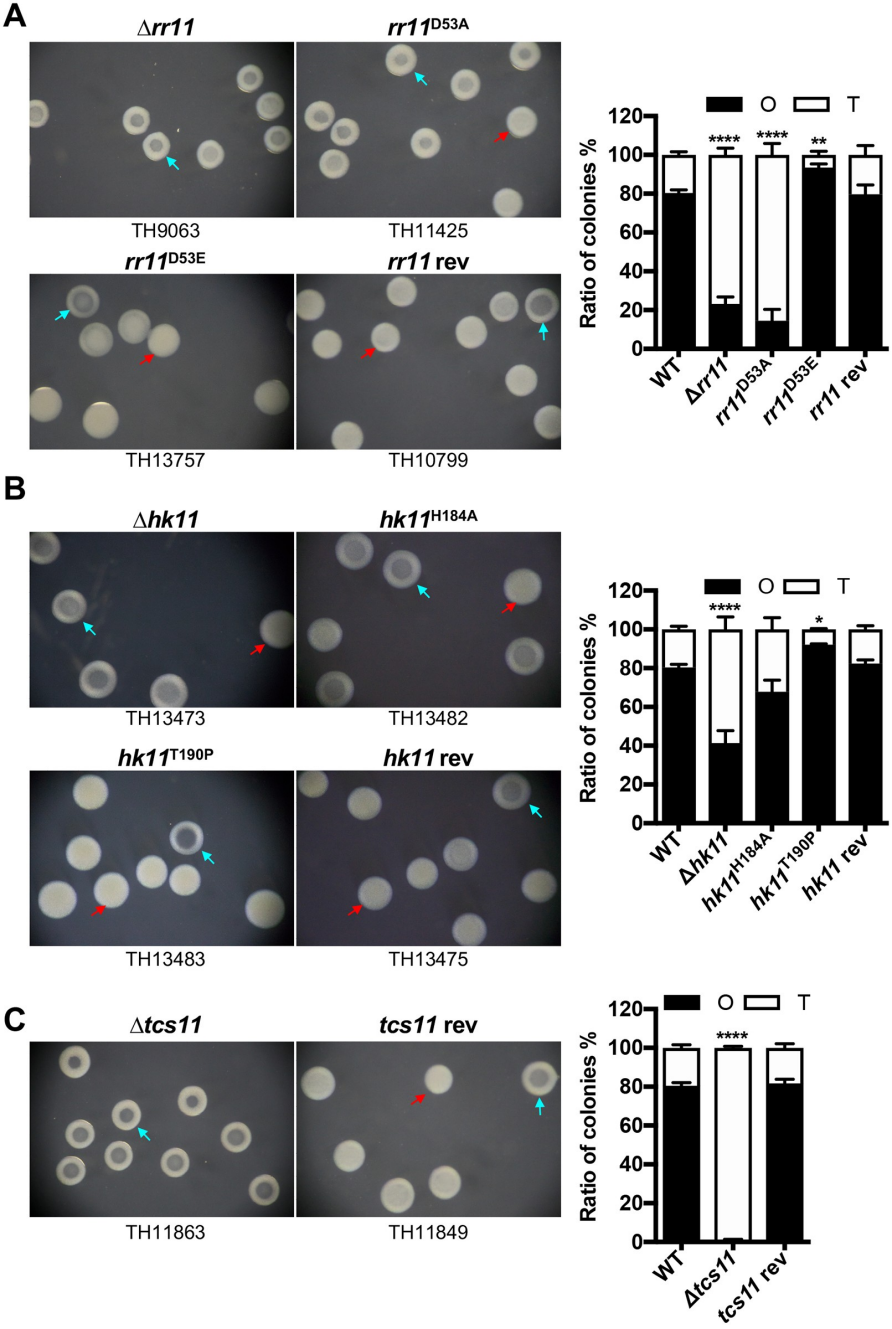
Colony opacity characteristics of the *tcs11* mutants. **A**. Colonies characteristics of the *rr11* mutants. The colony phenotypes of ST606 derivatives lacking *rr11* or carrying the D53A, D53E or wildtype *rr11* allele were characterized and presented essentially as in Fig 1. **B**. Colonies characteristics of the *hk11* mutants. Same as in A except for using ST606 derivatives with various *hk11* alleles. **C**. Colonies characteristics of the *tcs11* mutants. Same as in A except for using ST606 derivative lacking both the *rr11* and *hk11* of the 11^th^ two-component system (Δ*tcs11*) and *tcs11* revertant.

We further characterized the molecular mechanism behind the action of RR11 in regulation of pneumococcal phase variation. We first tested whether RR11 depends on phosphorylation state of the 53^rd^ aspartic acid residue (D^53^) for its role in phase variation by making a D-to-A point mutation at this site. D^53^ has been identified as an amino acid residue to receive phosphoryl group for activation of RR11 [43]. Similar to the *rr11* deletion mutant (TH9063), the *rr11*^D53A^ strain (TH11425) displayed a “hypo-O” phenotype, in which the proportion of O colonies decreased to 14.5% (Fig 5A). The effect of this point mutation on colony phenotype was successfully rescued by *in situ* replacing the *rr11*^D53A^ allele with the wildtype *rr11*, suggesting that phosphorylation of RR11 is required for its modulation of phase variation. Previous studies have found that the substitution of the conserved aspartic acid residue (D) for phosphorylation in bacterial response regulators with glutamic acid (E) can trigger proteins in an activated or “constitutively phosphorylated” state in the absence of the cognate sensing kinase [48, 49]. We thus constructed a ST606 derivative carrying a *rr11*^D53E^ allele by replacing the endogenous *rr11* in the original chromosomal locus (TH13757). Consistent with the predicted “constitutive phosphorylation” status of the RR11 protein, the *rr11*^D53E^ strain showed a “hyper-O” phenotype with 93.4% of O colonies in a clonally derived population (Fig 5A). Furthermore, reverting the *rr11* deletion with the wildtype *rr11* allele fully restored the colony phenotype of the mutant to that of the parental strain. The revertant produced 79.7% O colonies. These genetic and phenotypic characterizations indicated that the phosphorylated form of RR11 is required for the generation and/or maintenance of an O colony-dominated population.

We next tested the role of HK11, the sensor histidine kinase partner of RR11, in modulation of pneumococcal colony phenotype by unmarked deletion of *hk11* in ST606. The Δ*hk11* strain formed 41.4% O colonies, a significant reduction from that of the parental strain (80.3%), although it still produced more O colonies than the Δ*rr11* strain (23.2%) (Fig 5B). This result suggested that the HK11 is involved in phase variation. To verify this finding, we constructed a H-to-A mutation at the 184^th^ histidine residue of the HK11 because this represents the highly conserved site for auto-phosphorylation of sensing kinases in bacterial TCSs [13]. The *hk11*^H184A^ mutant showed only a modest reduction in proportion of O colonies (with 67.8% O colonies) although this reduction was fully restored in the *hk11* revertant (Fig 5B). The milder phenotype of the *hk11*^H184A^ strain than that of *rr11*^D53A^ suggested that RR11 is phosphorylated by an alternative phosphoryl group donor(s) besides HK11. Hentrich *et al*. reported that intracellular acetyl phosphate generated by pyruvate kinase SpxB is capable of phosphorylating the RR05 (CiaR) response regulator [50]. To elaborate on the function of HK11 in phase variation, we constructed a “hyper-active” mutant of HK11 by making a T-to-P mutation in the 190^th^ threonine residue. Previous study has demonstrated that the same mutation in the corresponding threonine residue at the 230^th^ position of HK05 (CiaH) renders the protein constitutively active as the sensor kinase [20]. The “constitutively active” *hk11*^T190P^ strain displayed a “hyper-O” phenotype (with 92.2% O colonies), which resembles the phenotype of the “constitutively phosphorylated” RR11 (*rr11*^D53E^) strain. These lines of evidence showed that the kinase activity of the sensing kinase HK11 is necessary for phosphorylation of RR11 in generation and/or maintenance of an O colony-dominated population.

Finally, we tested the phenotypic impact of the entire *tcs11* locus by simultaneous deletion of both the *rr11* and *hk11* genes in ST606. In a dramatic fashion, the *tcs11* mutant lost the ability to produce O colonies (0.4%) and produced only T colonies (Fig 5C). This deficiency was fully complemented to the parental level (81.6%) in the revertant. Taken together, the combinations of gene deletion and complementation unequivocally have demonstrated that the TCS11 system regulates the direction of phase variation in favor of the O phase by phosphorylation of the RR11 response regulator.

### Phosphorylated RR11 stabilizes the “opaque ON” genotype in a PsrA-dependent manner

**Fig 6:**
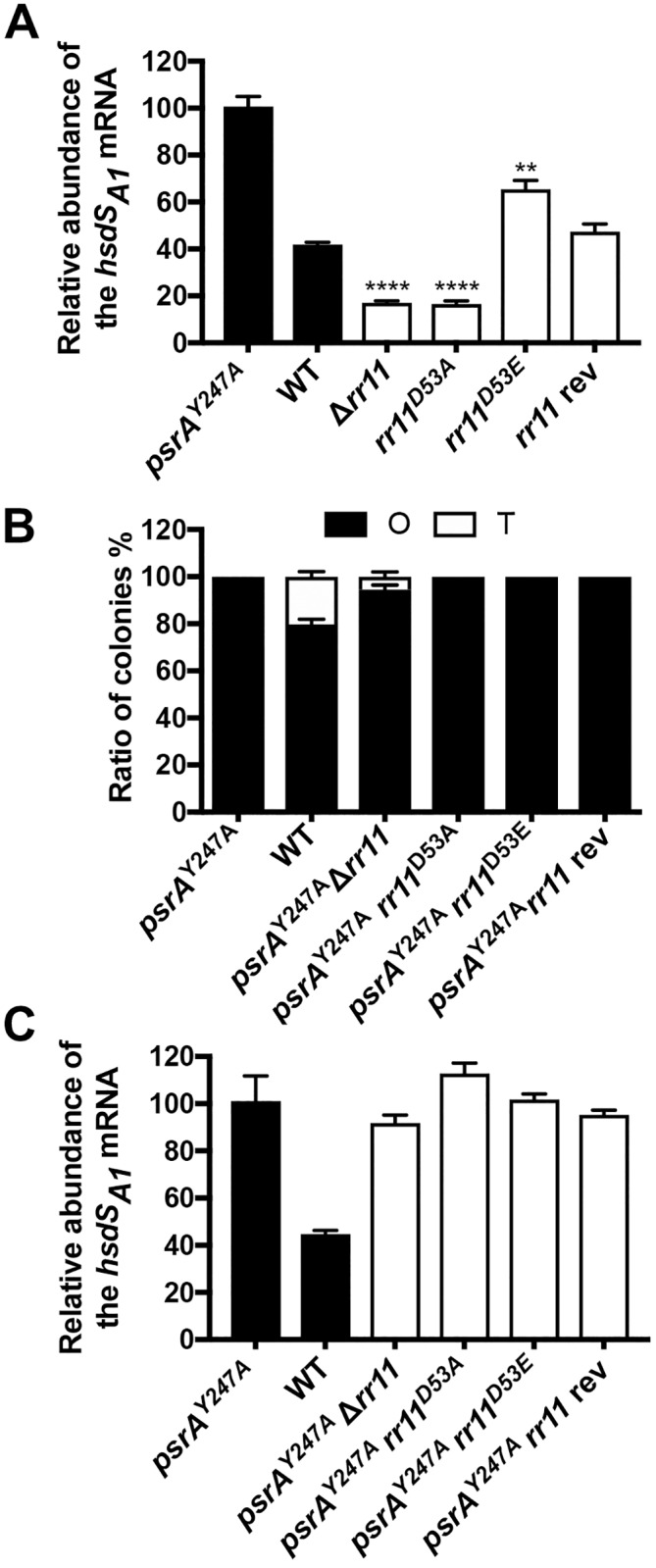
Impact of the *rr11* mutations on the *hsdS*_*A1*_ allelic configuration. **A**. Relative abundance of the *hsdS*_*A1*_ mRNA in the *rr11* mutants. The mRNA levels of the *hsdS*_*A1*_ allele in the clonal populations of ST606 (WT), isogenic Δ*rr11* and *psrA*^Y247A^ mutants were detected by qRT-PCR. The transcripts of the 5’ non-invertible segment of *hsdS*_*A*_ were similarly detected in all strains as a reference to calculate the relative *C*_*T*_ values. The relative *C*_*T*_ values of the ST606 and Δ*rr11* strains were then normalized to those of the *psrA*^Y247A^ strain that has the locked *hsdS*_*A1*_ allele (100%). The data are shown as mean ± SEM of a representative experiment. Each experiment was replicated at least twice. **B**. Colony opacity of the *psrA*^Y247A^-*rr11* double mutants. ST606 (WT) and its derivatives with either the *psrA*^Y247A^ allele alone or additional *rr11* allelic modifications were processed for colony enumeration, photographing and data presentation as in Fig 1B. **C**. Relative abundance of the *hsdS*_*A1*_ mRNA in the *psrA*^Y247A^-*rr11* double mutants. Same as in Fig 6A except for different strains.

Because only the *hsdS*_*A1*_ allele, one of the six functional *hsdS*_*A*_ alleles derived from the PsrA-driven inversions in the *cod* locus, enables pneumococci to produce O colonies [5, 6], the requirement of TCS11 for the O colony-dominant phenotype implied that this two-component system enriches the fraction of the *hsdS*_*A1*_-carrying variant in the population. We thus determined relative abundance of the *hsdS*_*A1*_-carrying variant in the populations of ST606 and various TCS11 mutants by measuring the relative mRNA abundance of the *hsdS*_*A1*_ allele in the context of the six *hsdS*_*A*_ allelic variants. The quantitative reverse transcriptase PCR (qRT-PCR) analysis showed 41.9% *hsdS*_*A1*_ allele out of the six potential *hsdS*_*A*_ alleles in the parental strain ST606 (Fig 6A). However, the *hsdS*_*A1*_ transcript was reduced to 17.1% and 16.5% in the *rr11*-null and *rr11*^D53A^ mutants, respectively, but the significantly decreased representation of the *hsdS*_*A1*_ allele in the RR11-deficient populations was restored to the parental level in the *rr11* revertant (*rr11* rev). Consistent with its “opaque ON” phenotype, the “hyper-phosphorylated” *rr11*^D53E^ mutant showed significantly increased *hsdS*_*A1*_ transcript (65.4%). This result indicated that phosphorylated RR11 enriches the *hsdS*_*A1*_ variant out of the six *hsdS*_*A*_ allelic variants.

We next determined if the function of RR11 in regulation of pneumococcal colony phases involves PsrA-catalyzed inversions in the *cod* locus by constructing multiple *rr11* mutations in the invertase-negative *psrA*^Y247A^ strain. In sharp contrast to remarkable impact of RR11 on the colony opacity phenotype in the wild type pneumococci (Fig 5), all genetic manipulations of *rr11* in the *psrA*^Y247A^ strain did not show obvious impact on the opaque-locked phenotype. All of the *rr11* mutants (Δ*rr11*, *rr11*^D53A^ and *rr11*^D53E^) in the *psrA*^Y247A^ background showed the “O” phenotype (Fig 6B; S3 Fig), indicating that RR11 is no longer able to impact the colony phases in the absence of the *hsdS* inversions. To verify the importance of PsrA in the regulation of RR11 on pneumococcal colony phases, we determined the fraction of the *hsdS*_*A1*_-carrying cells in various genetic backgrounds of *psrA* and *rr11* by measuring the relative abundance of the *hsdS*_*A1*_ mRNA molecules in these strains. As shown in Fig 6C, all of the three *rr11* mutants (Δ*rr11*, *rr11*^D53A^ and *rr11*^D53E^) generated in the *psrA*^Y247A^ background had a uniformly increased level of the *hsdS*_*A1*_ mRNAs as compared with the *psrA*^+^ ST606 strain. Taken together, these results have demonstrated that RR11 collaborates with the PsrA invertase in regulating the *hsdS*_*A*_ allelic configurations in the *cod* locus.

### Multiple RR11-regulated genes are required for stabilization of the O phase

To determine how RR11 regulates pneumococcal colony phases, we compared the transcriptomes between ST606 and Δ*rr11* mutant by RNA sequencing (RNA-seq). This trial identified a total of 23 genes with significant reduction (>2-fold change) in transcription between ST606 and Δ*rr11* (Table 3). This result thus revealed that RR11 acts as a transcriptional activator under these conditions. Except for *comW*, MYY691 and MYY1747, all the RR11-activated genes are concentrated in six apparent operons (Table 3; S4A Fig). While *comW* encodes the DNA-binding regulator ComW, a transcriptional activator of the competence genes [51, 52], virtually all of the other down-regulated genes in the *rr11* mutant are associated with substrate uptake and metabolism. However, the *rr11* mutant showed a similar level of the *psrA* mRNA as the parental strain (S4 Table). This result indicated that RR11 does not regulate pneumococcal colony phases through modulating the transcription of *psrA*.

**Table 3:** Differentially expressed genes in the *rr11* mutant.

Gene tag	Read count		Fold change	Products
	WT	Δ*rr11*		
MYY46	64	28	-2.1	Competence positive regulator ComW
MYY134	711	162	-4.0	Beta-galactosidase BgaC
MYY135	128	42	-2.8	Galactosamine-specific PTS IIB component GadV
MYY136	259	86	-2.8	Galactosamine-specific PTS IIC component GadW
MYY137	190	61	-2.9	Galactosamine-specific PTS IID component GadE
MYY138	171	76	-2.1	Galactosamine-specific PTS IIA component GadF
MYY139	340	145	-2.2	Galactosamine-6-phosphate isomerase AgaS
MYY404	203	81	-2.3	Unsaturated chondroitin disaccharide hydrolase Ugl
MYY405	80	34	-2.2	Hyaluronate-oligosaccharide-specific PTS IIB
MYY691	435	196	-2.0	Galactose-specific PTS IIC component GatC
MYY1048	215	80	-2.5	Lactose-specific PTS IIBC components LacE-2
MYY1049	161	54	-2.7	6-phospho-beta-galactosidase LacG-2
MYY1747	884	406	-2.0	Galactose-1-phosphate uridylyltransferase GalT-2
MYY1791	1516	650	-2.1	Sucrose phosphorylase GtfA
MYY1792	81	27	-2.8	Repeat region
MYY1793	518	210	-2.3	Multiple sugar ABC transporter, membrane-spanning permease protein MsmG (RafG)
MYY1794	627	219	-2.6	Multiple sugar ABC transporter, membrane-spanning permease protein MsmF (RafF)
MYY1795	1188	469	-2.3	Multiple sugar ABC transporter, substrate-binding protein MsmE (RafE)
MYY1796	1632	571	-2.6	Alpha-galactosidase AgaN (Aga)
MYY1924	210	62	-3.1	ABC transporter, permease protein
MYY1925	130	2	-59.7	ABC transporter, ATP-binding protein
MYY2067	840	338	-2.3	Arginine deiminase ArcA
MYY2068	767	304	-2.3	Ornithine carbamoyltransferase ArcB

**Fig 7:**
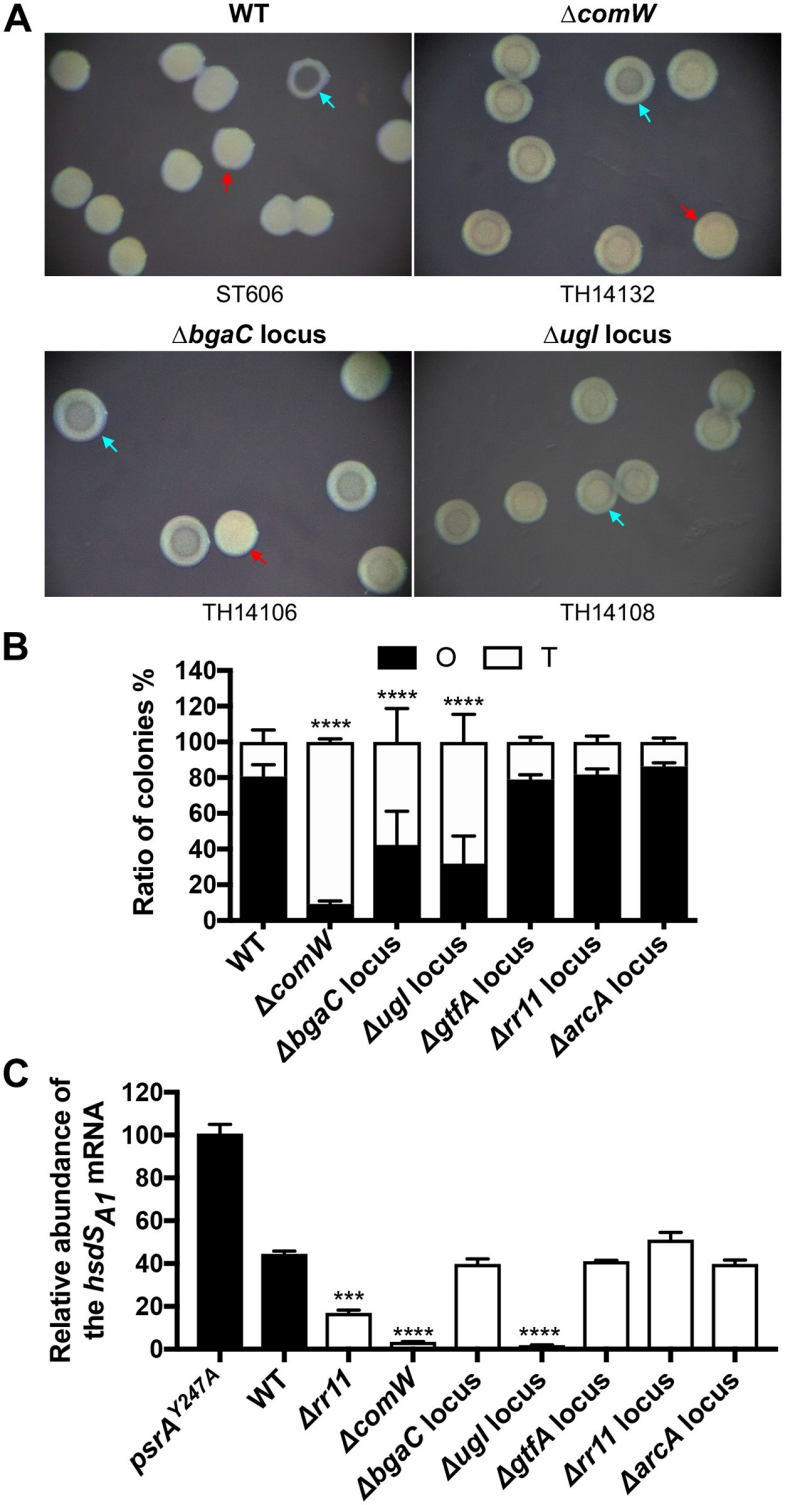
Impact of the RR11-regulated genes on the colony phases. **A**. Colony opacity characteristics of the RR11-regulated gene mutants. The O and T colonies were prepared and photographed as in Fig 1A. **B**. Relative composition of the O and T colony types in each strain were enumerated and presented as in Fig 1B. **C**. Relative abundance of the *hsdS*_*A1*_ mRNA in the populations of the RR11-regulated gene mutants. The mRNA levels of the *hsdS*_*A1*_ allele in ST606 (WT) and its mutant lacking RR11-regulated genes were detected, analyzed and presented in the same manner as in Fig 6A.

We next determined potential contribution of the RR11-regulated genes to the colony phases. The qRT-PCR result confirmed significant down-regulation of 24 genes within the RR11-regulated loci identified by the RNA-seq (S4B Fig). Further deletion analysis revealed significant shift in the colony phenotype in the mutants of three RR11-regulated gene loci (Fig 7A). The fraction of the O colonies in the *comW* mutant was dramatically diminished to 9.3% from 80.7% in the parental strain (Fig 7B). To a less extent, unmarked deletion of the genes in the two sugar phosphotransferase systems (PTS) also led to significant reduction in the fraction of the O colonies (Fig 7A). The mutants of the *bgaC* (MYY134-139) and *ugl* (MYY403-408) loci showed 42.4% and 31.9% O colonies, respectively (Fig 7B). While the *bgaC* locus is implicated in pneumococcal uptake and utilization of galactose and N-acetylgalactosamine [53, 54], the *ugl* gene cluster is necessary for uptake and metabolism of hyaluronic acid [55, 56]. The mutagenesis did not detect obvious changes in the mutants of the other RR11-regulated loci as exemplified by the ΔMYY1793-1796 (putative ABC transporter, *gtfA* locus), ΔMYY1924-1925 (putative ABC transporter, *rr11* locus) and ΔMYY2067-2068 (arginine metabolism, *arcA* locus) strains (Fig 7B). This result revealed that the competence regulator ComW and two sugar utilization systems are associated with pneumococcal phase variation.

To define how the RR11-regulated genes impact the colony phenotype, we tested if *comW* and the two sugar utilization loci are involved in the dominance of the *hsdS*_*A1*_-carrying cells in the ST606 population. Relative abundance of the *hsdS*_*A1*_ allele (out of the six potential *hsdS*_*A*_ allelic variants in the *cod* locus) was compared between ST606 and isogenic mutants of the RR11-regulated gene loci by testing the levels of the *hsdS*_*A1*_ mRNA using qRT-PCR. As compared with the invertase-negative *psrA*^Y247A^ strain (an *hsdS*_*A1*_-locked strain producing 100% *hsdS*_*A1*_ mRNA), the parental strain ST606 produced 44.5% *hsdS*_*A1*_ mRNA (Fig 7C). However, the level was reduced to 3.5% and 1.9% in the mutants of the *comW* and *ugl* loci, respectively, indicating that *comW* and the hyaluronate utilization locus are essential for stabilization of *hsdS*_*A1*_ allelic configuration in the *cod* locus. In sharp contrast to its significant change in colony phenotype (Fig 7B), the mutant of the *bgaC* locus had a comparable level of the *hsdS*_*A1*_ allele as the parental strain (Fig 7C). This result suggested that the galactose utilization system impacts pneumococcal colony phases in an inversion-independent manner. As represented by the ΔMYY1793-1796 (*gtfA* locus), ΔMYY1924-1925 (*rr11* locus) and ΔMYY2067-2068 (*arcA* locus) mutants, deleting other RR11-regulated genes did not lead to significant change in the allelic dominance of *hsdS*_*A1*_ in ST606 (Fig 7C). Taken together, these data showed that three of the RR11-regulated gene loci are necessary for stabilization of the O phase. While the c*omW* and hyaluronate utilization loci achieve this function by enforcing the *hsdS*_*A1*_ allelic configuration in the *cod* locus, the galactose utilization system impacts pneumococcal colony phases in an inversion-independent manner.

## Discussion

**Fig 8:**
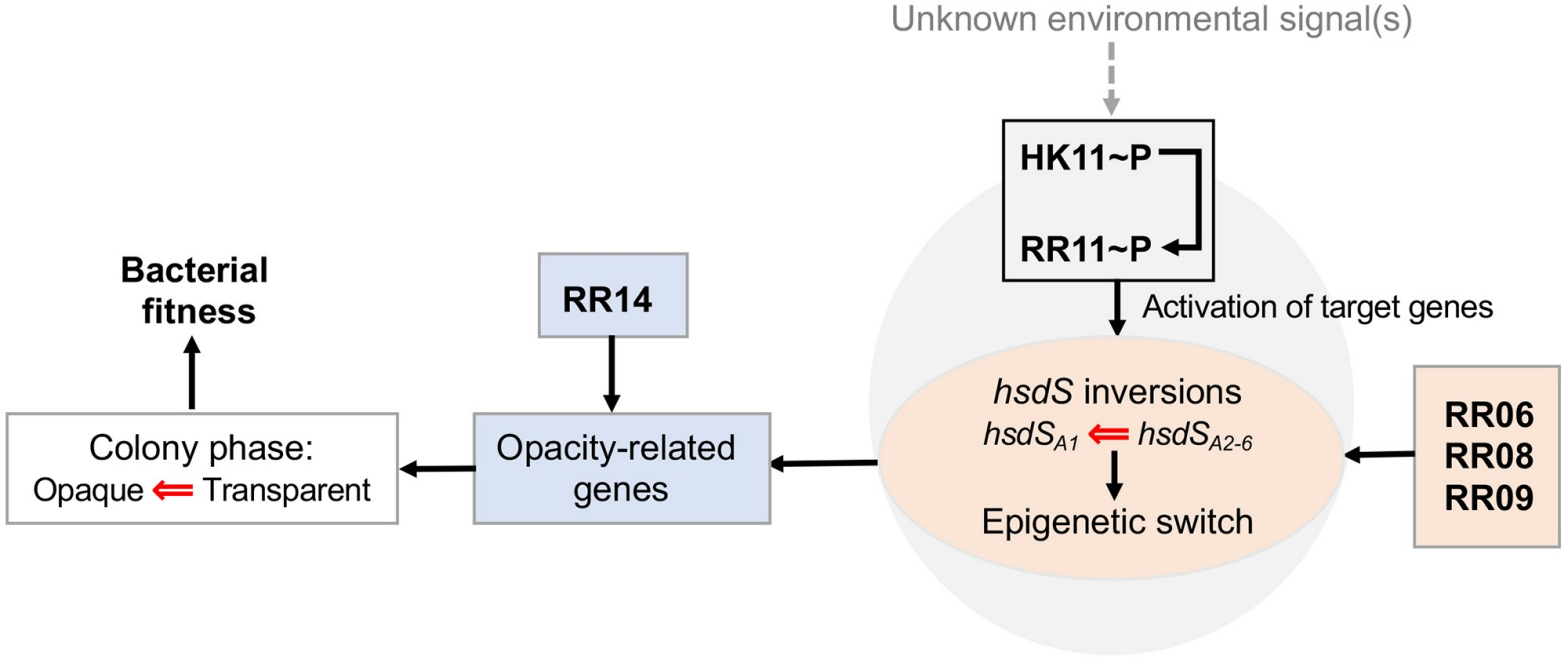
Working model depicting modulation of pneumococcal colony phases by the two-component systems. In response to unspecified environmental cue(s) sensed by HK11, the 53^rd^ conserved aspartic acid residue of RR11 is phosphorylated by HK11 or an alternative donor(s) of phosphoryl group, which in turn activates the c*omW* and hyaluronate utilization locus. By the undefined mechanism(s), the RR11-activated genes act to drive the directions of the *hsdS* inversions toward the *hsdS*_*A1*_ allelic configuration and eventually the O colony phase. Likewise, RR06, RR08 and RR09 promote the *hsdS*_*A1*_ allelic configuration and O colony phase by the uncharacterized mechanisms. RR14 modulates colony phases in a non-epigenetic manner.

Phase variation in colony opacity has been well characterized as an important strategy for pneumococcal adaption to various host environmental conditions [3, 4]. However, it is completely unknown whether the directions of the reversible switches between O and T colonies are re-balanced in response to environmental conditions. Our systematic screening of the 13 two-component response regulators, for the first time, has demonstrated that the balance between the O and T phases of *S*. *pneumoniae* is subjective to modulation by five TCSs in the *hsdS* inversion-dependent (RR06, RR08, RR09 and RR11) and -independent (RR14) manners. Although the precise environmental signal(s) sensed by each of the regulators remains to be identified, this study uncovers a new level of molecular sophistication and complexity in bacterial fine tuning of its biological activities and behaviors by linking the two-component environmental sensing systems to the epigenetic switching machinery (illustrated in Fig 8). Furthermore, the colony phenotypes of the TCS mutants identified in this work are highly valuable for systematic characterization of pneumococcal two-component systems in the future because their biological contributions remain largely unknown due to the lack of phenotypic hints.

This study has shown that, once being phosphorylated by its cognate sensing kinase HK11 and/or other phosphoryl group donor(s), RR11 indirectly enforces the O colony phase of *S*. *pneumoniae* by transcriptional activation of its target genes. The importance of RR11 phosphorylation is supported by the similarly diminished O phase of the *rr11*-null and phosphorylation deficient *rr11*^D53A^ strains. Consistently, the RR11 hyper-phosphorylation *rr11*^D53E^ strain displayed a hyper-O colony phenotype. Mutations in the key amino acid residues of the HK11 have also corroborated the significance of RR11 phosphorylation in favoring the O phase. Lastly, complete loss of the O colonies in the TCS11-deficient mutant has provided the striking phenotypic evidence for the essential role of this two-component system in modulating the directions of PsrA-mediated inversions and phase variation. More severe phenotype of the *tcs11*-null mutant (0.4% O colonies) than the *rr11* counterpart (reduced O colonies) suggests that HK11 may cross-phosphorylate other response regulators (e.g. RR06, RR08 and RR09) that are involved in modulating the directions of the inversion reactions in the *cod* locus. Cross-phosphorylation of bacterial response regulators has been well documented [57].

RR11 activates the transcription of *comW* and two sugar utilization gene (*bgaC* and *ugl*) loci that are necessary for the maintenance of the O colony phase. Among the 23 RR11-regulated genes identified by RNA-seq, *comW* and two sugar utilization gene (*bgaC* and *ugl*) loci are shown to promote the O phase. The c*omW* and hyaluronate utilization (*ugl*) loci accomplish this function by modulation of the PsrA-catalyzed inversions in the *cod* locus because deleting *comW* or the entire *ugl* locus not only led to significant loss of the O colonies but also diminished the dominance of the bacterial cells carrying the “opaque ON” *hsdS*_*A1*_ allele in the clonal populations. In contrast, loss of the galactose utilization (*bgaC*) system only shrank the representation of the O colonies without significant change in the status of *hsdS*_*A1*_ in the mutant population. This information points to the conclusion that the *bgaC* locus impacts pneumococcal colony phases in an inversion-independent manner. While it remains to be determined if all of the genes in the *ugl* and *bgaC* sugar utilization loci are involved in modulation of the colony phases, the existing information has provided more mechanistic hint for the action of ComW.

ComW may regulate *hsdS* inversions through its physical interactions with unknown enhancer sequence(s) and/or PsrA. ComW is known as a competence-specific protein that stabilizes and activates the alternative sigma factor ComX for transcriptional activation of the natural transformation genes [51, 52]. A recent study shows that ComW binds DNA independent of DNA sequence, and its activity in activating pneumococcal transformation depends on the DNA binding activity [58], but this protein has not been associated with any non-competence function. In the context, ComW could act like the HU and/or Fis DNA-binding proteins of *Salmonella enterica* serovar Typhimurium. HU and Fis (factor for inversion stimulation) are the nucleoid-associated proteins that promote the Hin recombinase-catalyzed inversions, the best-characterized DNA inversion system in prokaryotes [59, 60]. The Hin-mediated inversions of a ~1 kb sequence flanked by the two *hix* sites (inverted repeat sequences recognized by the Hin recombinase) lead to alternative expression of the FljB and FliC flagellins and thereby drives phase (antigenic) variation in two different flagellar serotypes [59, 61]. While HU loops the invertible sequence [60, 62, 63], Fis enhances assembly of the supercoiling-dependent invertasome by binding to a 65-bp enhancer sequence (within the invertible sequence) [64]. The Fis/enhancer complex also determines the direction and rotation of the Hin synaptic complex [65]. It is possible that ComW enhances the formation and activity of the PsrA invertasome through its DNA-binding activity. Alternatively, binding of ComW to its target sequence(s) may regulate the *hsdS* inversions by altering the supercoiling state of the local sequences. It has been shown that negative DNA supercoiling is essential for the Hin-mediated inversions [60, 66].

Multiple lines of experimental evidence have also revealed that RR06, RR08 and RR09 promote the O colony phase by modulating the direction of the PsrA-catalyzed *hsdS* inversions. The first set of the proof came from the impact of the *psrA* gene on the colony phenotypes of the *rr06*, *rr08* and *rr09* mutants. Although these mutants generated in the *psrA*^+^ genetic background showed significantly reduced proportions of O colonies, the phase-locked O counterpart derived from the PsrA-deficient strain uniformly produced O colonies. Consistent with their reduced fractions of O colonies, the *rr06*, *rr08* and *rr09* mutants also exhibited diminished methylation for the DNA motif recognized by the “opaque ON” HsdS_A1_ MTase. Although the current data cannot explain how the pneumococci utilize multiple response regulators for regulation of *hsdS* inversion reactions, these factors must act in a highly coordinated manner in the context of environmental/cellular conditions. As exemplified with TCS11, further identification of the genes regulated by RR06, RR08 and RR09 under these conditions will be necessary for elucidation of the molecular mechanisms governing the actions of these TCSs in pneumococcal phase variation.

The existing information strongly suggests that pneumococcal phase variation in colony opacity is a gross reflection of pneumococcal physiological conditions, such as redox and metabolism. The linkage between pneumococcal colony phases and redox conditions is supported by significant impact of the orphan response regulator RR14 (RitR) on the colony phases. RR14 represses the transcription of the pneumococcal iron uptake (Piu) and nutrient uptake loci [39]. A recent study has shown that RR14 serves as a principal regulator in response to oxidative stress via the cysteine-mediated dimerization, which enhances pneumococcal tolerance to hydrogen peroxide and other oxidants [40]. *S*. *pneumoniae* is famous for its production of millimolar range of hydrogen peroxide as a metabolic byproduct of pyruvate oxidase SpxB [67, 68]. Consistently, SpxB is under-expressed in the opaque colony variant [69]. Potential association of pneumococcal metabolism with colony phases agrees with the known regulation of the nutrient uptake systems by RR14 [39]. Moreover, significant impact of the *bgaC* and *ugl* sugar utilization systems on the O phase could be explained by their roles in cellular metabolism. Lastly, broad impact of the five TCSs on the colony phases implies that the appearance of pneumococcal colonies is defined by vastly different mechanisms/pathways.

## Materials and methods

### Bacterial strains and reagents

The bacterial strains used in this work are listed in S5 Table. *S*. *pneumoniae* was grown in Todd-Hewitt broth with 0.5% yeast extract (THY) or on tryptic soy agar (TSA) plate as described [70]. Streptomycin (150 μg/ml) and kanamycin (400 μg/ml) were added in the medium when necessary. All chemical reagents used in this study were purchased from Sigma (Shanghai, China) unless otherwise indicated. DNA processing enzymes were purchased from New England Biolabs (Beijing, China). All primers were synthesized by Synbio Tech (Beijing, China) and are listed in S6 Table. All Sanger sequencing data were obtained from Ruibiotech company (Beijing, China).

### Construction of pneumococcal mutants

Pneumococcal mutants were constructed in streptomycin-resistant strains ST606 (derivative of ST556, serotype 19F), TH6671 (derivative of P384, serotype 6A) and TH6675 (derivative of ST877, serotype 35B) essentially as described [5]. DNA templates, primers, restriction enzymes, resulting strains, genotypes and other details associated with mutant construction are listed in S7 Table. Briefly, JC1 (a modified Janus cassette used in our previous studies) replacement of the target sequences was generated as followed: the up- and down-stream arms of each target sequence were PCR amplified, digested with XbaI and XhoI, and ligated to a XbaI/XhoI-digested JC1 before being transformed into target pneumococcal strains. JC1 was amplified by primers Pr9840 and Pr1098 from genomic DNA of strain TH7919 (TH5445Δ*bgaA*::JC1) [5]. Unmarked deletions were subsequently constructed in the JC1 replacement strains by transformation with either ligation or fusion PCR products of the up- and down-stream target sequences.

The genetic revertants were constructed by transformation of JC1 replacement strains with amplicon of the corresponding wildtype target genes from genomic DNA of ST606. Gene deletions in the *psrA*^Y247A^ background were similarly constructed in strain TH6552 [5]. The point mutations in *rr11* and *hk11* were generated by introducing the mutations in the primers used for fusion PCR. Specifically, the codon of D53 (GAT) in *rr11* was converted to GCG (A) and GAG (E), respectively; the codons of H184 (CAT) and T190 (ACC) in *hk11* to GCC (A) and CCT (P), respectively. The *bgaC*, *ugl*, *gtfA*, *rr11* and *arcA* locus deletion mutants were constructed by unmarked deletion of MYY134-139, MYY403-408, MYY1793-1796, MYY1924-1925 and MYY2067-2068 as indicated in S7 Table, respectively. Mutated sequence in each strain was confirmed by PCR amplification and DNA sequencing.

### Microscopic quantification of O and T colonies

Pneumococcal colony opacity was microscopically assessed with colonies grown on catalase-TSA plates (6,000 units catalase/9-cm diameter plate) under 37 °C, 5% CO_2_ for 17 hrs as described [71]. Each inoculum was adjusted according to its OD_620nm_ value to yield ~200 colonies per plate for quantification of the O and T colonies for each strain. The ratio between the two types of colonies for each strain was obtained with triplicate plates each time, and subsequently repeated at least twice. The ratio of the progeny colonies generated from individual O and T seeding colonies was determined as previously described [5].

### SMRT sequencing

Methylation of pneumococcal genome was assessed by SMRT sequencing essentially as described [5]. Genomic DNA was isolated from colonies grown on catalase-TSA plates using the HiPure Bacterial DNA Kit (Magen, Beijing, China) according to the manufacturer’s protocol. Briefly, pneumococci were grown on the plates under 37 °C, 5% CO_2_ for 17 hrs before the colonies were scraped off with a glass spreader and washed once with pre-chilled Ringer’s solution. Bacterial lysis was achieved in 250 μl Buffer STE supplemented with 10 μl of 10% sodium deoxycholate (DOC) solution before the lysates were processed to remove RNA and proteins. The final DNA extracts were eluted with 35 μl of deionized distilled water and quantified with the NanoDrop 2000 spectrophotometer (Thermo Scientific, USA). 10Kb SMRT Bell library was constructed and sequenced using the PacBio RSII sequencing platform at the Novogene Bioinformatics Technology (Beijing, China). The low-quality reads were filtered by the SMRT Link v5.0.1 and the filtered reads was subsequently assembled by SMRT portal (Version 2.3.0) to generate one contig without gaps. The raw data for the results presented in this work are available at the NCBI database under the following accessions: SRR10083119 (ST606, wild type), SRR10083118 (TH9048 Δ*rr01*), SRR10083113 (TH7009 Δ*rr03*), SRR10083112 (TH9054 Δ*rr04*), SRR10083111 (TH10784 Δ*rr05*), SRR10083110 (TH9164 Δ*rr06*), SRR10083109 (TH9057 Δ*rr07*), SRR10083108 (TH9181 Δ*rr08*), SRR10083107 (TH8468 Δ*rr09*), SRR10083106 (TH9060 Δ*rr10*), SRR10083117 (TH9063 Δ*rr11*), SRR10083116 (TH9259 Δ*rr12*), SRR10083115 (TH9066 Δ*rr13*) and SRR10083114 (TH9167 Δ*rr14*).

### RNA-seq

RNA sequencing (RNA-seq) was carried out as described with minor modifications [19]. Specifically, pneumococci were cultured on catalase-TSA plates for 17 hrs (a time for the routine colony photographing) before colonies on multiple plates were collected in pre-chilled Ringer’s solution and pooled as described for genomic DNA purification. Bacteria in suspensions were pelleted by centrifugation at 4 °C, frozen in liquid nitrogen and stored at -80 °C. Total RNA was extracted from the frozen samples with the Purelink^™^ RNA Mini Kit (Invitrogen, USA) and further purified with Qiagen RNeasy MinElute spin columns according to the manufacturer’s protocol (Qiagen, Germany). RNA-seq was performed at the Novogene Bioinformatics Technology (Beijing, China). Trimmed reads were mapped to the genome of *S*. *pneumoniae* ST556 (CP003357.2) using Bowtie 2.3.1 and Tophat 2.1.1. Significant difference was defined by an at least 2-fold change and a *P* value of < 0.001. All of the raw RNA-seq data presented in this work are available in NCBI’s Gene Expression Omnibus (GEO) database (accession GSE137447). The result of each sample represents the means of two independent experiments.

### qRT-PCR

Pneumococcal mRNAs were quantified by quantitative real-time reverse transcriptase PCR (qRT-PCR) as described [72]. Briefly, total RNA extracts were prepared as described for RNA-seq and used to prepare cDNA pools with iScript^™^ cDNA Synthesis Kit (Bio-Rad, USA). The 367-bp *hsdS*_*A1*_ allele-specific sequence was amplified with primers Pr16174/Pr16175. As an internal reference for PCR, the 5’ non-invertible region shared by the six *hsdS*_*A*_ alleles was also amplified using primers Pr16178/Pr16179. The relative abundance of *hsdS*_*A1*_ mRNA was obtained by a two-tier approach. The Δ*C*_T_ value for each strain was firstly calculated by subtracting the average *C*_T_ value of the non-invertible *hsdS*_*A*_ reactions from the *C*_T_ values of the *hsdS*_*A1*_-specific reactions. Because the *hsdS*_*A1*_-specific sequence (367 bp) was substantially longer than the common *hsdS*_*A*_ region (267 bp), the Δ*C*_T_ value of each strain was further normalized to the counterpart of *psrA*^*Y247A*^ by subtracting the average Δ*C*_T_ of *psrA*^*Y247A*^ from that of each strain. Our previous study showed that the loss-of-function mutation in *psrA* made the *psrA*^*Y247A*^ strain genetically locked in the *hsdS*_*A1*_ allelic state [5], and should produce only the *hsdS*_*A1*_ mRNA. The relative abundance of *hsdS*_*A1*_ mRNA of each strain is presented as (2^-ΔΔ*C*T^) % given that the relative abundance of *hsdS*_*A1*_ mRNA in *psrA*^*Y247A*^ is 100%. The transcriptional levels of RR11-regulated genes were detected by qRT-PCR with the *era* gene as an internal control and primers listed in S8 Table. The *era* gene was amplified with primers Pr7932/Pr7933, which is commonly used as an internal control [72]. The relative gene expression was calculated according to the comparative 2^-ΔΔ*C*T^ method [73] and the ΔΔ*C*_T_ was calculated using the following equation: ΔΔ*C*_T_ = (*C*_T_ gene of interest -

*C*_T_ *era*) mutant-(*C*_T_ gene of interest-*C*_T_ *era*) ST606. The data from one representative experiment are presented as mean value of triplicate samples ± SEM (standard error of mean) for each strain. Each experiment was repeated at least twice.

### Statistical analysis

The colony ratio data was statistically analyzed by two-sided Chi-square test (means); qRT-PCR, relative abundance of *hsdS*_*A1*_ mRNA data by two-tailed unpaired Student’s *t* test. The relevant data are presented as mean ± SEM. Significant differences are defined by *P* values of < 0.05 (*), < 0.01 (**), < 0.001(***) and < 0.0001 (****).

## Supporting information

S1 Table The opacity ratio of six regulator mutants and their isogenic revertants. (DOCX)

S2 Table Methylation sequences specified by the Spn556I MTase. (DOCX)

S3 Table Methylation sequences specified by the Spn556III MTases. (DOCX)

S4 Table The transcripts of Δ*rr11* mutant. (XLSX)

S5 Table Bacterial strains used in this study. (DOCX)

S6 Table Primers used in this study. (DOCX)

S7 Table PCR amplifications used for pneumococcal mutagenesis in this study. (DOCX)

S8 Table The qRT-PCR settings in this study. (DOCX)

S1 Fig Colony opacity of the isogenic revertants of *rr* mutants. ST606 *rr* isogenic revertants were grown and processed for photographing of the colonies, and marked as in Fig 1A.
(TIF)

S2 Fig Colony opacity of the *psrA*^Y247A^-*rr* double mutants. ST606 derivatives with either the inactive *psrA*^Y247A^ allele alone (TH6552) or both the *psrA*^Y247A^ allele and unmarked deletion of a single *rr* gene were grown and processed for photographing of the colonies, and marked as in Fig 1A.
(TIF)

S3 Fig Colony opacity of the *psrA*^Y247A^-*rr11* double mutants. ST606 derivatives with either the inactive *psrA*^Y247A^ allele alone (TH6552) or both the *psrA*^Y247A^ allele and *rr11* mutants were grown and processed for photographing of the colonies, and marked as in Fig 1A.
(TIF)

S4 Fig The genetic organization and transcriptional expression of RR11-regulated gene loci. **A**. The genetic organization of the six RR11-regulated gene loci. The translational orientations of the genes in the six RR11-regulated gene loci are indicated with arrowheads; each gene identified with its functional or genomic names below; the number of nucleotides between two adjacent genes marked at the intergenic region. The genes deleted for mutagenesis were shown in bold. **B**. Transcription of the RR11-regulated genes in the Δ*rr11* mutant. Transcriptions of *comW* and the genes in the loci of MYY134-139, MYY403-408, MYY1791-1796, MYY1923-1925 and MYY2067-2068 in the ST606 (WT) and Δ*rr11* strains were detected by qRT-PCR. Relative transcriptional difference of each gene in the *rr11* mutant is calculated by normalizing the *C*_*T*_ value of each gene to that of the parental strain.
(TIF)

## References

[1] Bogaert D, de Groot R, Hermans PWM. *Streptococcus pneumoniae* colonisation: the key to pneumococcal disease. Lancet Infect Dis. 2004;4(3):144–54. 10.1016/S1473-3099(04)00938-7 14998500

[2] Weiser JN. Phase variation in colony opacity by *Streptococcus pneumoniae*. Microb Drug Resist. 1998;4(2):129–35. 10.1089/mdr.1998.4.129 . 9651000

[3] Weiser JN, Austrian R, Sreenivasan PK, Masure HR. Phase variation in pneumococcal opacity: relationship between colonial morphology and nasopharyngeal colonization. Infect Immun. 1994;62(6):2582–9. . 8188381 10.1128/iai.62.6.2582-2589.1994 PMC186548

[4] Kim JO, Weiser JN. Association of intrastrain phase variation in quantity of capsular polysaccharide and teichoic acid with the virulence of *Streptococcus pneumoniae*. J Infect Dis. 1998;177(2):368–77. 10.1086/514205 9466523

[5] Li J, Li JW, Feng Z, Wang J, An H, Liu Y, et al Epigenetic switch driven by DNA inversions dictates phase variation in *Streptococcus pneumoniae*. PLoS Pathog. 2016;12(7):e1005762 10.1371/journal.ppat.1005762 . 27427949 PMC4948785

[6] Li J, Zhang JR. Phase variation of *Streptococcus pneumoniae*. Microbiol Spectr. 2019;7(1):GPP3-0005-2018. 10.1128/microbiolspec.GPP3-0005-2018 . 30737916 PMC11590436

[7] Manso AS, Chai MH, Atack JM, Furi L, De Ste Croix M, Haigh R, et al A random six-phase switch regulates pneumococcal virulence via global epigenetic changes. Nat Commun. 2014;5:5055 10.1038/ncomms6055 . 25268848 PMC4190663

[8] De Ste Croix M, Vacca I, Kwun MJ, Ralph JD, Bentley SD, Haigh R, et al Phase-variable methylation and epigenetic regulation by type I restriction-modification systems. FEMS Microbiol Rev. 2017;41(Supp_1):S3–S15. 10.1093/femsre/fux025 . 28830092

[9] Li JW, Li J, Wang J, Li C, Zhang JR. Molecular mechanisms of *hsdS* inversions in the *cod* locus of *Streptococcus pneumoniae*. J Bacteriol. 2019;201(6):e00581–18. 10.1128/JB.00581-18 . 30617241 PMC6398273

[10] De Ste Croix M, Chen KY, Vacca I, Manso AS, Johnston C, Polard P, et al Recombination of the phase-variable *spnIII* locus is independent of all known pneumococcal site-specific recombinases. J Bacteriol. 2019;201(15)e00233–19. 10.1128/JB.00233-19 . 31085693 PMC6620402

[11] Loenen WAM, Dryden DTF, Raleigh EA, Wilson GG. Type I restriction enzymes and their relatives. Nucleic Acids Res. 2014;42(1):20–44. 10.1093/nar/gkt847 24068554 PMC3874165

[12] Stock AM, Robinson VL, Goudreau PN. Two-component signal transduction. Annu Rev Biochem. 2000;69:183–215. 10.1146/annurev.biochem.69.1.183 . 10966457

[13] Stock J, Park P, Surette M, Levit M. Two-component signal transduction system: structure-function relationship and mechanisms of catalysis 1995; p. 25–51. In: Hoch J, Silhavy T (ed), Two-Component Signal Transduction. ASM Press, Washington, DC 10.1128/9781555818319.ch3

[14] Lange R, Wagner C, de Saizieu A, Flint N, Molnos J, Stieger M, et al Domain organization and molecular characterization of 13 two-component systems identified by genome sequencing of *Streptococcus pneumoniae*. Gene. 1999;237(1):223–34. 10.1016/s0378-1119(99)00266-8 . 10524254

[15] Throup JP, Koretke KK, Bryant AP, Ingraham KA, Chalker AF, Ge Y, et al A genomic analysis of two-component signal transduction in *Streptococcus pneumoniae*. Mol Microbiol. 2000;35(3):566–76. 10.1046/j.1365-2958.2000.01725.x . 10672179

[16] Gomez-Mejia A, Gamez G, Hammerschmidt S. *Streptococcus pneumoniae* two-component regulatory systems: The interplay of the pneumococcus with its environment. Int J Med Microbiol. 2018;308(6):722–37. 10.1016/j.ijmm.2017.11.012 . 29221986

[17] Trihn M, Ge X, Dobson A, Kitten T, Munro CL, Xu P. Two-component system response regulators involved in virulence of *Streptococcus pneumoniae* TIGR4 in infective endocarditis. PLoS One. 2013;8(1):e54320 10.1371/journal.pone.0054320 . 23342132 PMC3546988

[18] Schnorpfeil A, Kranz M, Kovacs M, Kirsch C, Gartmann J, Brunner I, et al Target evaluation of the non-coding csRNAs reveals a link of the two-component regulatory system CiaRH to competence control in *Streptococcus pneumoniae* R6. Mol Microbiol. 2013;89(2):334–49. 10.1111/mmi.12277 . 23710838

[19] Liu Y, Zeng Y, Huang Y, Gu L, Wang S, Li C, et al HtrA-mediated selective degradation of DNA uptake apparatus accelerates termination of pneumococcal transformation. Mol Microbiol. 2019 10.1111/mmi.14364 . 31396996

[20] Guenzi E, Gasc AM, Sicard MA, Hakenbeck R. A two-component signal-transducing system is involved in competence and penicillin susceptibility in laboratory mutants of *Streptococcus pneumoniae*. Mol Microbiol. 1994;12(3):505–15. 10.1111/j.1365-2958.1994.tb01038.x . 8065267

[21] Sebert ME, Patel KP, Plotnick M, Weiser JN. Pneumococcal HtrA protease mediates inhibition of competence by the CiaRH two-component signaling system. J Bacteriol. 2005;187(12):3969–79. 10.1128/JB.187.12.3969-3979.2005 15937159 PMC1151733

[22] Pinas GE, Cortes PR, Orio AG, Echenique J. Acidic stress induces autolysis by a CSP-independent ComE pathway in *Streptococcus pneumoniae*. Microbiology. 2008;154(Pt 5):1300–8. 10.1099/mic.0.2007/015925-0 . 18451038

[23] Mascher T, Heintz M, Zahner D, Merai M, Hakenbeck R. The CiaRH system of *Streptococcus pneumoniae* prevents lysis during stress induced by treatment with cell wall inhibitors and by mutations in pbp2x involved in beta-lactam resistance. J Bacteriol. 2006;188(5):1959–68. 10.1128/JB.188.5.1959-1968.2006 . 16484208 PMC1426552

[24] Dagkessamanskaia A, Moscoso M, Henard V, Guiral S, Overweg K, Reuter M, et al Interconnection of competence, stress and CiaR regulons in *Streptococcus pneumoniae*: competence triggers stationary phase autolysis of *ciaR* mutant cells. Mol Microbiol. 2004;51(4):1071–86. 10.1111/j.1365-2958.2003.03892.x 14763981

[25] Salvadori G, Junges R, Morrison DA, Petersen FC. Competence in *Streptococcus pneumoniae* and close commensal relatives: mechanisms and implications. Front Cell Infect Microbiol. 2019;9:94 10.3389/fcimb.2019.00094 . 31001492 PMC6456647

[26] Pestova EV, Havarstein LS, Morrison DA. Regulation of competence for genetic transformation in *Streptococcus pneumoniae* by an auto-induced peptide pheromone and a two-component regulatory system. Mol Microbiol. 1996;21(4):853–62. 10.1046/j.1365-2958.1996.501417.x . 8878046

[27] Havarstein LS, Gaustad P, Nes IF, Morrison DA. Identification of the streptococcal competence-pheromone receptor. Mol Microbiol. 1996;21(4):863–9. 10.1046/j.1365-2958.1996.521416.x 8878047

[28] Gutu AD, Wayne KJ, Sham LT, Winkler ME. Kinetic characterization of the WalRKSpn (VicRK) two-component system of *Streptococcus pneumoniae*: dependence of WalKSpn (VicK) phosphatase activity on its PAS domain. J Bacteriol. 2010;192(9):2346–58. 10.1128/JB.01690-09 . 20190050 PMC2863487

[29] Wayne KJ, Sham LT, Tsui HC, Gutu AD, Barendt SM, Keen SK, et al Localization and cellular amounts of the WalRKJ (VicRKX) two-component regulatory system proteins in serotype 2 *Streptococcus pneumoniae*. J Bacteriol. 2010;192(17):4388–94. 10.1128/JB.00578-10 . 20622066 PMC2937396

[30] Eldholm V, Gutt B, Johnsborg O, Bruckner R, Maurer P, Hakenbeck R, et al The pneumococcal cell envelope stress-sensing system LiaFSR is activated by murein hydrolases and lipid II-interacting antibiotics. J Bacteriol. 2010;192(7):1761–73. 10.1128/JB.01489-09 . 20118250 PMC2838051

[31] Zheng JJ, Sinha D, Wayne KJ, Winkler ME. Physiological roles of the dual phosphate transporter systems in low and high phosphate conditions and in capsule maintenance of *Streptococcus pneumoniae* D39. Front Cell Infect Microbiol. 2016;6:63 10.3389/fcimb.2016.00063 . 27379215 PMC4913102

[32] Novak R, Cauwels A, Charpentier E, Tuomanen E. Identification of a *Streptococcus pneumoniae* gene locus encoding proteins of an ABC phosphate transporter and a two-component regulatory system. J Bacteriol. 1999;181(4):1126–33. 9973337 10.1128/jb.181.4.1126-1133.1999 PMC93488

[33] Standish AJ, Stroeher UH, Paton JC. The two-component signal transduction system RR06/HK06 regulates expression of *cbpA* in *Streptococcus pneumoniae*. Proc Natl Acad Sci U S A. 2005;102(21):7701–6. 10.1073/pnas.0409377102 . 15897461 PMC1140415

[34] Ma Z, Zhang JR. RR06 activates transcription of *spr1996* and *cbpA* in *Streptococcus pneumoniae*. J Bacteriol. 2007;189(6):2497–509. 10.1128/JB.01429-06 . 17220227 PMC1899362

[35] McKessar SJ, Hakenbeck R. The two-component regulatory system TCS08 is involved in cellobiose metabolism of *Streptococcus pneumoniae* R6. J Bacteriol. 2007;189(4):1342–50. 10.1128/JB.01170-06 17028271 PMC1797370

[36] de Saizieu A, Gardes C, Flint N, Wagner C, Kamber M, Mitchell TJ, et al Microarray-based identification of a novel *Streptococcus pneumoniae* regulon controlled by an autoinduced peptide. J Bacteriol. 2000;182(17):4696–703. 10.1128/jb.182.17.4696-4703.2000 . 10940007 PMC111343

[37] Reichmann P, Hakenbeck R. Allelic variation in a peptide-inducible two-component system of *Streptococcus pneumoniae*. Fems Microbiol Lett. 2000;190(2):231–6. 10.1111/j.1574-6968.2000.tb09291.x 11034284

[38] Kjos M, Miller E, Slager J, Lake FB, Gericke O, Roberts IS, et al Expression of *Streptococcus pneumoniae* bacteriocins is induced by antibiotics via regulatory interplay with the competence system. PLoS Pathog. 2016;12(2):e1005422 10.1371/journal.ppat.1005422 . 26840404 PMC4739728

[39] Ulijasz AT, Andes DR, Glasner JD, Weisblum B. Regulation of iron transport in *Streptococcus pneumoniae* by RitR, an orphan response regulator. J Bacteriol. 2004;186(23):8123–36. 10.1128/JB.186.23.8123-8136.2004 . 15547286 PMC529065

[40] Glanville DG, Han L, Maule AF, Woodacre A, Thanki D, Abdullah IT, et al RitR is an archetype for a novel family of redox sensors in the streptococci that has evolved from two-component response regulators and is required for pneumococcal colonization. PLoS Pathog. 2018;14(5):e1007052 10.1371/journal.ppat.1007052 . 29750817 PMC5965902

[41] Blue CE, Mitchell TJ. Contribution of a response regulator to the virulence of *Streptococcus pneumoniae* is strain dependent. Infect Immun. 2003;71(8):4405–13. 10.1128/IAI.71.8.4405-4413.2003 12874319 PMC166049

[42] Hendriksen WT, Silva N, Bootsma HJ, Blue CE, Paterson GK, Kerr AR, et al Regulation of gene expression in *Streptococcus pneumoniae* by response regulator 09 is strain dependent. J Bacteriol. 2007;189(4):1382–9. 10.1128/JB.01144-06 17085554 PMC1797359

[43] Park AK, Lee JH, Chi YM, Park H. Structural characterization of the full-length response regulator spr1814 in complex with a phosphate analogue reveals a novel conformational plasticity of the linker region. Biochem Biophys Res Commun. 2016;473(2):625–9. 10.1016/j.bbrc.2016.03.144 . 27038544

[44] Tsui HC, Zheng JJ, Magallon AN, Ryan JD, Yunck R, Rued BE, et al Suppression of a deletion mutation in the gene encoding essential PBP2b reveals a new lytic transglycosylase involved in peripheral peptidoglycan synthesis in *Streptococcus pneumoniae* D39. Mol Microbiol. 2016;100(6):1039–65. 10.1111/mmi.13366 . 26933838 PMC5063045

[45] Lukat GS, McCleary WR, Stock AM, Stock JB. Phosphorylation of bacterial response regulator proteins by low molecular weight phospho-donors. Proc Natl Acad Sci U S A. 1992;89(2):718–22. 10.1073/pnas.89.2.718 . 1731345 PMC48310

[46] Feng Z, Li J, Zhang JR, Zhang X. qDNAmod: a statistical model-based tool to reveal intercellular heterogeneity of DNA modification from SMRT sequencing data. Nucleic Acids Res. 2014;42(22):13488–99. 10.1093/nar/gku1097 . 25404133 PMC4267614

[47] Kwun MJ, Oggioni MR, De Ste Croix M, Bentley SD, Croucher NJ. Excision-reintegration at a pneumococcal phase-variable restriction-modification locus drives within- and between-strain epigenetic differentiation and inhibits gene acquisition. Nucleic Acids Res. 2018;46(21):11438–53. 10.1093/nar/gky906 30321375 PMC6265443

[48] Smith JG, Latiolais JA, Guanga GP, Pennington JD, Silversmith RE, Bourret RB. A search for amino acid substitutions that universally activate response regulators. Mol Microbiol. 2004;51(3):887–901. 10.1046/j.1365-2958.2003.03882.x . 14731287

[49] Martin B, Soulet AL, Mirouze N, Prudhomme M, Mortier-Barriere I, Granadel C, et al ComE/ComE~P interplay dictates activation or extinction status of pneumococcal X-state (competence). Mol Microbiol. 2013;87(2):394–411. 10.1111/mmi.12104 . 23216914

[50] Hentrich K, Lofling J, Pathak A, Nizet V, Varki A, Henriques-Normark B. *Streptococcus pneumoniae* senses a human-like sialic acid profile via the response regulator CiaR. Cell Host Microbe. 2016;20(3):307–17. 10.1016/j.chom.2016.07.019 . 27593514 PMC5025396

[51] Sung CK, Morrison DA. Two distinct functions of ComW in stabilization and activation of the alternative sigma factor ComX in *Streptococcus pneumoniae*. J Bacteriol. 2005;187(9):3052–61. 10.1128/JB.187.9.3052-3061.2005 . 15838032 PMC1082825

[52] Luo P, Li H, Morrison DA. Identification of ComW as a new component in the regulation of genetic transformation in *Streptococcus pneumoniae*. Mol Microbiol. 2004;54(1):172–83. 10.1111/j.1365-2958.2004.04254.x . 15458414

[53] Jeong JK, Kwon O, Lee YM, Oh DB, Lee JM, Kim S, et al Characterization of the *Streptococcus pneumoniae* BgaC protein as a novel surface beta-galactosidase with specific hydrolysis activity for the Galbeta1-3GlcNAc moiety of oligosaccharides. J Bacteriol. 2009;191(9):3011–23. 10.1128/JB.01601-08 . 19270088 PMC2681812

[54] Afzal M, Shafeeq S, Ahmed H, Kuipers OP. N-acetylgalatosamine-mediated regulation of the *aga* operon by AgaR in *Streptococcus pneumoniae*. Front Cell Infect Microbiol. 2016;6:101 10.3389/fcimb.2016.00101 . 27672623 PMC5018945

[55] Maruyama Y, Nakamichi Y, Itoh T, Mikami B, Hashimoto W, Murata K. Substrate specificity of streptococcal unsaturated glucuronyl hydrolases for sulfated glycosaminoglycan. J Biol Chem. 2009;284(27):18059–69. 10.1074/jbc.M109.005660 . 19416976 PMC2709336

[56] Marion C, Stewart JM, Tazi MF, Burnaugh AM, Linke CM, Woodiga SA, et al *Streptococcus pneumoniae* can utilize multiple sources of hyaluronic acid for growth. Infect Immun. 2012;80(4):1390–8. 10.1128/IAI.05756-11 22311922 PMC3318431

[57] Agrawal R, Sahoo BK, Saini DK. Cross-talk and specificity in two-component signal transduction pathways. Future Microbiol. 2016;11(5):685–97. 10.2217/fmb-2016-0001 27159035

[58] Inniss NL, Prehna G, Morrison DA. The pneumococcal sigma(X) activator, ComW, is a DNA-binding protein critical for natural transformation. J Biol Chem. 2019;294(29):11101–18. 10.1074/jbc.RA119.007571 . 31160340 PMC6643036

[59] Johnson RC. Site-specific DNA inversion by serine recombinases. Microbiol Spectr. 2015;3(1):MDNA3-0047-2014. 10.1128/microbiolspec.MDNA3-0047-2014 . 26104558

[60] Johnson RC, Bruist MF, Simon MI. Host protein requirements for in vitro site-specific DNA inversion. Cell. 1986;46(4):531–9. 10.1016/0092-8674(86)90878-0 . 3524854

[61] Zieg J, Silverman M, Hilmen M, Simon M. Recombinational switch for gene expression. Science. 1977;196(4286):170–2. 10.1126/science.322276 . 322276

[62] Hillyard DR, Edlund M, Hughes KT, Marsh M, Higgins NP. Subunit-specific phenotypes of Salmonella typhimurium HU mutants. J Bacteriol. 1990;172(9):5402–7. 10.1128/jb.172.9.5402-5407.1990 . 2168381 PMC213205

[63] Haykinson MJ, Johnson RC. DNA looping and the helical repeat in vitro and in vivo: effect of HU protein and enhancer location on Hin invertasome assembly. EMBO J. 1993;12(6):2503–12. 10.1002/j.1460-2075.1993.tb05905.x . 8508775 PMC413488

[64] McLean MM, Chang Y, Dhar G, Heiss JK, Johnson RC. Multiple interfaces between a serine recombinase and an enhancer control site-specific DNA inversion. Elife. 2013;2:e01211 10.7554/eLife.01211 24151546 PMC3798978

[65] Dhar G, Heiss JK, Johnson RC. Mechanical constraints on Hin subunit rotation imposed by the Fis/enhancer system and DNA supercoiling during site-specific recombination. Mol Cell. 2009;34(6):746–59. 10.1016/j.molcel.2009.05.020 . 19560425 PMC2752211

[66] Johnson RC, Bruist MB, Glaccum MB, Simon MI. In vitro analysis of Hin-mediated site-specific recombination. Cold Spring Harb Symp Quant Biol. 1984;49:751–60. 10.1101/sqb.1984.049.01.085 . 6099257

[67] Pericone CD, Park S, Imlay JA, Weiser JN. Factors contributing to hydrogen peroxide resistance in *Streptococcus pneumoniae* include pyruvate oxidase (SpxB) and avoidance of the toxic effects of the fenton reaction. J Bacteriol. 2003;185(23):6815–25. 10.1128/JB.185.23.6815-6825.2003 . 14617646 PMC262707

[68] Lisher JP, Tsui HT, Ramos-Montanez S, Hentchel KL, Martin JE, Trinidad JC, et al Biological and chemical adaptation to endogenous hydrogen peroxide production in *Streptococcus pneumoniae* D39. mSphere. 2017;2(1). 10.1128/mSphere.00291-16 . 28070562 PMC5214746

[69] Overweg K, Pericone CD, Verhoef GG, Weiser JN, Meiring HD, De Jong AP, et al Differential protein expression in phenotypic variants of *Streptococcus pneumoniae*. Infect Immun. 2000;68(8):4604–10. 10.1128/iai.68.8.4604-4610.2000 . 10899862 PMC98388

[70] Chen H, Ma Y, Yang J, O’Brien CJ, Lee SL, Mazurkiewicz JE, et al Genetic requirement for pneumococcal ear infection. Plos One. 2008;3(8):e2950 10.1371/journal.pone.0002950 18670623 PMC2593789

[71] Li J, Wang J, Jiao F, Zhang J-R. Observation of pneumococcal phase variation in colony morphology. Bio-protocol. 2017;7(15):e2434 10.21769/BioProtoc.2434 PMC8413639 34541155

[72] Liu X, Li JW, Feng Z, Luo Y, Veening JW, Zhang JR. Transcriptional repressor PtvR regulates phenotypic tolerance to vancomycin in *Streptococcus pneumoniae*. J Bacteriol. 2017;199(14). 10.1128/JB.00054-17 . 28484041 PMC5494751

[73] Schmittgen T. D. Livak K. J. Analyzing real-time PCR data by the comparative *C*_T_ method. Nat Protoc. 2008;3(6):1101–8. 10.1038/nprot.2008.73 18546601

