# Supplementary material for: Regulation of pneumococcal epigenetic and colony phases by multiple two-component regulatory systems
Source: PLoS Pathog. 2020 Mar 18;16(3):e1008417. doi: 10.1371/journal.ppat.1008417 (PMC7105139; doi:10.1371/journal.ppat.1008417)
Supplement: S1 Table — (DOCX) [file ppat.1008417.s001.docx]

**Table S1. The opacity ratio of six regulator mutants and its isogenic revertants^1^**

| **Strain (genotype)** | **No. of colonies** | | **%^2^ opaque** | **%^3^ transparent** | **SEM (±)^4^** | **Chi-square, df** | ***P* value** | **Colony phenotype** |
| --- | --- | --- | --- | --- | --- | --- | --- | --- |
|  | **Opaque** | **Transparent** |  |  |  |  |  |  |
| ST606 (ST556 *rpsL1*) | 92 | 26 | 77.5 | 22.5 | 0.9 | **_** | **_** | Normal |
|  | 118 | 32 |  |  |  |  |  |  |
|  | 78 | 25 |  |  |  |  |  |  |
| TH10784 (Δ*rr05*) | 84 | 60 | 60.8 | 39.2 | 1.7 | 5.984, 1 | 0.0144 | Transparent |
|  | 63 | 42 |  |  |  |  |  |  |
|  | 80 | 45 |  |  |  |  |  |  |
| TH10790 (*rr05* rev) | 131 | 0 | 100.0 | 0 | 0 | 25.99, 1 | <0.0001 | Opaque |
|  | 120 | 0 |  |  |  |  |  |  |
|  | 109 | 0 |  |  |  |  |  |  |
| TH9164 (Δ*rr06*) | 12 | 140 | 11.5 | 88.5 | 2.5 | 85.53, 1 | <0.0001 | Transparent |
|  | 13 | 114 |  |  |  |  |  |  |
|  | 20 | 102 |  |  |  |  |  |  |
| TH9553 (*rr06* rev) | 100 | 28 | 74.1 | 25.9 | 2.8 | 0.2433, 1 | 0.6218 | Normal |
|  | 110 | 50 |  |  |  |  |  |  |
|  | 117 | 38 |  |  |  |  |  |  |
| TH9181 (Δ*rr08*) | 48 | 87 | 30.6 | 69.4 | 4.4 | 42.59, 1 | <0.0001 | Transparent |
|  | 45 | 86 |  |  |  |  |  |  |
|  | 34 | 121 |  |  |  |  |  |  |
| TH10793 (*rr08* rev) | 121 | 30 | 71.2 | 28.8 | 7.0 | 0.9356, 1 | 0.3334 | Normal |
|  | 62 | 46 |  |  |  |  |  |  |
|  | 76 | 24 |  |  |  |  |  |  |
| TH8468 (Δ*rr09*) | 43 | 97 | 31.9 | 68.1 | 1.5 | 40.83, 1 | <0.0001 | Transparent |
|  | 47 | 88 |  |  |  |  |  |  |
|  | 37 | 86 |  |  |  |  |  |  |
| TH10796 (*rr09* rev) | 92 | 23 | 81.1 | 18.9 | 1.1 | 0.4822, 1 | 0.4874 | Normal |
|  | 80 | 20 |  |  |  |  |  |  |
|  | 90 | 18 |  |  |  |  |  |  |
| TH9063 (Δ*rr11*) | 50 | 139 | 23.7 | 76.3 | 1.8 | 56.19, 1 | <0.0001 | Transparent |
|  | 45 | 140 |  |  |  |  |  |  |
|  | 31 | 122 |  |  |  |  |  |  |
| TH10799 (*rr11* rev) | 104 | 37 | 74.9 | 25.1 | 5.1 | 0.1096, 1 | 0.7405 | Normal |
|  | 96 | 18 |  |  |  |  |  |  |
|  | 80 | 40 |  |  |  |  |  |  |
| TH9167 (Δ*rr14*) | 24 | 128 | 15.5 | 84.5 | 0.7 | 74.79, 1 | <0.0001 | Transparent |
|  | 20 | 120 |  |  |  |  |  |  |
|  | 24 | 121 |  |  |  |  |  |  |
| TH9555 (*rr14* rev) | 70 | 50 | 67.2 | 32.8 | 5.1 | 2.48, 1 | 0.1153 | Normal |
|  | 74 | 36 |  |  |  |  |  |  |
|  | 76 | 24 |  |  |  |  |  |  |

^1^the opaque and transparent colonies on each plate in a representative experiment were enumerated as described in Fig. 1.

^2, 3^ the average opaque and transparent colony ratio were calculated by the average of followed values from three plates: number of each form of colony divided by the total colony number *100%.

^4^The standard error of means were calculated on the basis of the three duplicate plates.
