# Supplementary material for: Regulation of pneumococcal epigenetic and colony phases by multiple two-component regulatory systems
Source: PLoS Pathog. 2020 Mar 18;16(3):e1008417. doi: 10.1371/journal.ppat.1008417 (PMC7105139; doi:10.1371/journal.ppat.1008417)
Supplement: S2 Table — (DOCX) [file ppat.1008417.s002.docx]

**Table S2. Methylation sequences specified by the Spn556I MTase^1^**

| **Genotype** | **5’-TCTAG^m6^A-3’**  **3’-^m6^AGATCT-5’** | | |
| --- | --- | --- | --- |
|  | # in  genome^2^ | #  detected^3^ | %  detected^4^ |
| Wild type | 664 | 664 | 100 |
| ∆*rr01* | 664 | 664 | 100 |
| ∆*rr03* | 664 | 664 | 100 |
| ∆*rr04* | 664 | 664 | 100 |
| ∆*rr05* | 664 | 664 | 100 |
| ∆*rr06* | 664 | 653 | 98.3 |
| ∆*rr07* | 664 | 662 | 99.7 |
| ∆*rr08* | 664 | 652 | 98.2 |
| ∆*rr09* | 664 | 649 | 97.7 |
| ∆*rr10* | 664 | 664 | 100 |
| ∆*rr11* | 664 | 656 | 98.8 |
| ∆*rr12* | 664 | 656 | 98.8 |
| ∆*rr13* | 664 | 664 | 100 |
| ∆*rr14* | 664 | 664 | 100 |

^1^The accumulative number of all methylated loci in each strain exceeded 100% because a base was considered as being methylated once more than 30% of all the reads at the position passed the cutoff value in the PacBio platform.

^2^Total number of loci in both DNA strands in the genome of ST556 (accession CP003357.2).

^3^Total loci detected by the SMRT sequencing.

^4^Percentage of the detected motifs was calculated as follows: total loci detected/total loci in the genome.
