# Supplementary material for: Regulation of pneumococcal epigenetic and colony phases by multiple two-component regulatory systems
Source: PLoS Pathog. 2020 Mar 18;16(3):e1008417. doi: 10.1371/journal.ppat.1008417 (PMC7105139; doi:10.1371/journal.ppat.1008417)
Supplement: S3 Table — (DOCX) [file ppat.1008417.s003.docx]

**Table S3. Methylation sequences specified by the Spn556III MTases^1^**

| **Genotype** | **HsdS_1_** | | | **HsdS_2_** | | | **HsdS_3_** | | |
| --- | --- | --- | --- | --- | --- | --- | --- | --- | --- |
|  | **5’-GAT^m6^AN_7_TCA-3’**  **3’-CTATN_7_^m6^AGT-5’** | | | **5’-GG^m6^AN_7_TGA-3’**  **3’-CCTN_7_^m6^ACT-5’** | | | **5’-GG^m6^AN_7_TCA-3’**  **3’-CCTN_7_^m6^AGT-5’** | | |
|  | # in  genome^2^ | # detected^3^ | %  detected^4^ | # in genome | # detected | % detected | # in genome | # detected | % detected |
| Wild type | 1246 | 1244 | 99.8 | 2244 | 798 | 35.6 | 2080 | 0 | 0 |
| ∆*rr01* | 1246 | 1246 | 100 | 2244 | 0 | 0 | 2080 | 0 | 0 |
| ∆*rr03* | 1246 | 1246 | 100 | 2244 | 0 | 0 | 2080 | 0 | 0 |
| ∆*rr04* | 1246 | 1246 | 100 | 2244 | 0 | 0 | 2080 | 0 | 0 |
| ∆*rr05* | 1246 | 1246 | 100 | 2244 | 0 | 0 | 2080 | 0 | 0 |
| ∆*rr06* | 1246 | 1210 | 97.1 | 2244 | 0 | 0 | 2080 | 0 | 0 |
| ∆*rr07* | 1246 | 1241 | 99.6 | 2244 | 0 | 0 | 2080 | 0 | 0 |
| ∆*rr08* | 1246 | 1198 | 96.1 | 2244 | 0 | 0 | 2080 | 0 | 0 |
| ∆*rr09* | 1246 | 753 | 60.4 | 2244 | 0 | 0 | 2080 | 0 | 0 |
| ∆*rr10* | 1246 | 1246 | 100 | 2244 | 0 | 0 | 2080 | 0 | 0 |
| ∆*rr11* | 1246 | 1226 | 98.4 | 2244 | 0 | 0 | 2080 | 0 | 0 |
| ∆*rr12* | 1246 | 1223 | 98.2 | 2244 | 0 | 0 | 2080 | 0 | 0 |
| ∆*rr13* | 1246 | 0 | 0 | 2244 | 0 | 0 | 2080 | 2077 | 99.9 |
| ∆*rr14* | 1246 | 1243 | 99.8 | 2244 | 0 | 0 | 2080 | 0 | 0 |

^1^The accumulative number of all methylated loci in each strain exceeded 100% because a base was considered as being methylated once more than 30% of all the reads at the position passed the cutoff value in the PacBio platform.

^2^Total number of loci in both DNA strands in the genome of ST556 (accession CP003357.2).

^3^Total loci detected by the SMRT sequencing.

^4^Percentage of the detected motifs was calculated as follows: total loci detected/total loci in the genome.

N, any nucleotide.
