# Supplementary material for: Regulation of pneumococcal epigenetic and colony phases by multiple two-component regulatory systems
Source: PLoS Pathog. 2020 Mar 18;16(3):e1008417. doi: 10.1371/journal.ppat.1008417 (PMC7105139; doi:10.1371/journal.ppat.1008417)
Supplement: S5 Table — (DOCX) [file ppat.1008417.s005.docx]

**Table S5. Bacterial strains used in this study**

| Strain | Description | Reference or source |
| --- | --- | --- |
| ST556 | *Streptococcus pneumoniae* strain, serotype 19F, encapsulated | (1) |
| ST606 | ST556 derivative; *rpsL1* | (1) |
| TH6552 | TH5993 derivative; *psrA*^Y247A^ | (2) |
| TH6671 | *Streptococcus pneumoniae* strain, serotype 6A, P384 derivative; *rpsL1* | (1) |
| TH6675 | *Streptococcus pneumoniae* strain, serotype 35B, ST877 derivative; *rpsL1* | (1) |
| TH6986 | ST606 derivative; ∆*rr03*::JC1 | This study |
| TH7009 | TH6986 derivative; ∆*rr03* | This study |
| TH7919 | TH5445 Δ*bgaA*::JC1 | (1) |
| TH8425 | ST606 derivative; ∆*rr09*::JC1 | This study |
| TH8468 | TH8425 derivative; ∆*rr09* | This study |
| TH9025 | ST606 derivative; ∆*rr01*::JC1 | This study |
| TH9029 | ST606 derivative; ∆*rr04*::JC1 | This study |
| TH9031 | ST606 derivative; ∆*rr07*::JC1 | This study |
| TH9033 | ST606 derivative; ∆*rr10*::JC1 | This study |
| TH9035 | ST606 derivative; ∆*rr11*::JC1 | This study |
| TH9037 | ST606 derivative; ∆*rr13*::JC1 | This study |
| TH9048 | TH9025 derivative; ∆*rr01* | This study |
| TH9054 | TH9029 derivative; ∆*rr04* | This study |
| TH9057 | TH9031 derivative; ∆*rr07* | This study |
| TH9060 | TH9033 derivative; ∆*rr10* | This study |
| TH9063 | TH9035 derivative; ∆*rr11* | This study |
| TH9066 | TH9037 derivative; ∆*rr13* | This study |
| TH9114 | ST606 derivative; ∆*rr06*::JC1 | This study |
| TH9115 | ST606 derivative; ∆*rr06*::JC1 | This study |
| TH9116 | ST606 derivative; ∆*rr12*::JC1 | This study |
| TH9118 | ST606 derivative; ∆*rr14*::JC1 | This study |
| TH9164 | TH9114 derivative; ∆*rr06* | This study |
| TH9167 | TH9118 derivative; ∆*rr14* | This study |
| TH9180 | ST606 derivative; ∆*rr08*::JC1 | This study |
| TH9181 | TH9180 derivative; ∆*rr08* | This study |
| TH9259 | TH9116 derivative; ∆*rr12* | This study |
| TH9553 | TH9115 derivative; *rr06* rev | This study |
| TH9555 | TH9116 derivative; *rr14* rev | This study |
| TH10415 | ST606 derivative; ∆*rr05*::JC1 | This study |
| TH10784 | TH10415 derivative; ∆*rr05* | This study |
| TH10790 | TH10415 derivative; *rr05* rev | This study |
| TH10793 | TH9180 derivative; *rr08* rev | This study |
| TH10796 | TH8425 derivative; *rr09* rev | This study |
| TH10799 | TH9035 derivative; *rr11* rev | This study |
| TH11403 | ST606 derivative; ∆*tcs11*::JC1 | This study |
| TH11425 | TH9035 derivative; *rr11*^D53A^ | This study |
| TH11849 | TH11403 derivative; *tcs11* rev | This study |
| TH11863 | TH11403 derivative; ∆*tcs11* | This study |
| TH13068 | TH6552 derivative; *psrA*^Y247A^ ∆*rr06*::JC1 | This study |
| TH13070 | TH6552 derivative; *psrA*^Y247A^ ∆*rr08*::JC1 | This study |
| TH13072 | TH6552 derivative; *psrA*^Y247A^ ∆*rr09*::JC1 | This study |
| TH13074 | TH6552 derivative; *psrA*^Y247A^ ∆*rr14*::JC1 | This study |
| TH13076 | TH6552 derivative; *psrA*^Y247A^ ∆*rr11*::JC1 | This study |
| TH13115 | TH13068 derivative; *psrA*^Y247A^ ∆*rr06* | This study |
| TH13117 | TH13070 derivative; *psrA*^Y247A^ ∆*rr08* | This study |
| TH13119 | TH13072 derivative; *psrA*^Y247A^ ∆*rr09* | This study |
| TH13121 | TH13074 derivative; *psrA*^Y247A^ ∆*rr11* | This study |
| TH13123 | TH13076 derivative; *psrA*^Y247A^ ∆*rr14* | This study |
| TH13125 | TH13076 derivative; *psrA*^Y247A^ *rr11*^D53A^ | This study |
| TH13127 | TH13076 derivative; *psrA*^Y247A^ *rr11* rev | This study |
| TH13418 | ST606 derivative; ∆MYY1924-1925::JC1 | This study |
| TH13428 | TH13418 derivative; ∆MYY1924-1925(*rr11* locus) | This study |
| TH13455 | ST606 derivative; ∆*hk11*::JC1 | This study |
| TH13473 | TH13455 derivative; ∆*hk11* | This study |
| TH13475 | TH13455 derivative; *hk11* rev | This study |
| TH13482 | TH13455 derivative; *hk11*^H184A^ | This study |
| TH13483 | TH13455 derivative; *hk11*^T190P^ | This study |
| TH13757 | TH9035 derivative; *rr11*^D53E^ | This study |
| TH13845 | TH13076 derivative; *psrA*^Y247A^ *rr11*^D53E^ | This study |
| TH13971 | TH6675 derivative; ST877 ∆*rr06*::JC1 | This study |
| TH13973 | TH6675 derivative; ST877 ∆*rr08*::JC1 | This study |
| TH13975 | TH6675 derivative; ST877 ∆*rr09*::JC1 | This study |
| TH13977 | TH6675 derivative; ST877 ∆*rr10*::JC1 | This study |
| TH13979 | TH6675 derivative; ST877 ∆*rr11*::JC1 | This study |
| TH13981 | TH6671 derivative; P384 ∆*rr06*::JC1 | This study |
| TH13983 | TH6671 derivative; P384 ∆*rr08*::JC1 | This study |
| TH13985 | TH6671 derivative; P384 ∆*rr09*::JC1 | This study |
| TH13987 | TH6671 derivative; P384 ∆*rr10*::JC1 | This study |
| TH13989 | TH6671 derivative; P384 ∆*rr11*::JC1 | This study |
| TH13998 | TH13971 derivative; ST877 ∆*rr06* | This study |
| TH14000 | TH13973 derivative; ST877 ∆*rr08* | This study |
| TH14002 | TH13975 derivative; ST877 ∆*rr09* | This study |
| TH14004 | TH13977 derivative; ST877 ∆*rr10* | This study |
| TH14006 | TH13979 derivative; ST877 ∆*rr11* | This study |
| TH14008 | TH13981 derivative; P384 ∆*rr06* | This study |
| TH14010 | TH13983 derivative; P384 ∆*rr08* | This study |
| TH14012 | TH13985 derivative; P384 ∆*rr09* | This study |
| TH14014 | TH13987 derivative; P384 ∆*rr10* | This study |
| TH14016 | TH13989 derivative; P384 ∆*rr11* | This study |
| TH14092 | ST606 derivative; ∆MYY134-139::JC1 | This study |
| TH14095 | ST606 derivative; ∆MYY2067-2068::JC1 | This study |
| TH14097 | ST606 derivative; ∆MYY403-408::JC1 | This study |
| TH14105 | ST606 derivative; ∆MYY1793-1796::JC1 | This study |
| TH14106 | TH14092 derivative; ∆MYY134-139(*bgaC* locus) | This study |
| TH14108 | TH14097 derivative; ∆MYY403-408(*ugl* locus) | This study |
| TH14110 | TH14095 derivative; ∆MYY2067-2068(*arcA* locus) | This study |
| TH14114 | ST606 derivative; ∆*comW*::JC1 | This study |
| TH14132 | TH14114 derivative; ∆*comW* | This study |
| TH14193 | TH14105 derivative; ∆MYY1793-1796(*gtfA* locus) | This study |

1. Li J, Li J-W, Feng Z, Wang J, An H, Liu Y, Wang Y, Wang K, Zhang X, Miao Z, Liang W, Sebra R, Wang G, Wang W-C, Zhang J-R. 2016. Epigenetic Switch Driven by DNA Inversions Dictates Phase Variation in Streptococcus pneumoniae. Plos Pathogens 12.

2. Li JW, Li J, Wang J, Li C, Zhang JR. 2019. MOLECULAR MECHANISMS OF HSDS INVERSIONS IN THE COD LOCUS OF STREPTOCOCCUS PNEUMONIAE. J Bacteriol doi:10.1128/jb.00581-18.
