# Supplementary material for: Regulation of pneumococcal epigenetic and colony phases by multiple two-component regulatory systems
Source: PLoS Pathog. 2020 Mar 18;16(3):e1008417. doi: 10.1371/journal.ppat.1008417 (PMC7105139; doi:10.1371/journal.ppat.1008417)
Supplement: S6 Table — (DOCX) [file ppat.1008417.s006.docx]

**Table S6. Primers used in this study**

| Primers | Sequence (5’-3’) |
| --- | --- |
| Pr1098 | GAGACTCGAGCCTTTCCTTATGCTTTTGGAC |
| Pr7932 | GATTGCCATCATGAGTGACAAGG |
| Pr7933 | AGTGTCCACTTCGCGAAGGGT |
| Pr9840 | GAGATCTAGAGGATAATGCTGAAAACTCCTTGAAG |
| Pr10418 | GAGGCAATCTTCTTGGACATATCGC |
| Pr10419 | GAGATCTAGAGGATGATTTAGCAGACTCATACTTTTCAGC |
| Pr10420 | GAGACTCGAGTCTAGAGAACCAAGCCATCGATGTTGAGG |
| Pr10421 | TCACGGTATTGGACGCTAGGAAGG |
| Pr10454 | GGAAGAGGAAATCATCGAGAAAGAACG |
| Pr10455 | GAGATCTAGAGTAGCTTTTCAAACCCAAACGGACC |
| Pr10456 | GAGATCTAGAGTAGCTTTTCAAACCCAAACGGACC |
| Pr10457 | TAGTCTCGCAGAGTCCAGCCAATG |
| Pr11507 | GGAGTATAACCCTGACCTGTCTCGATGTA |
| Pr11508 | GAGATCTAGACGTCAGTTTAAGAAGGGAGAAGGCC |
| Pr11509 | GAGACTCGAGGATTGTGTAGGTCATGATCTGCTCCTTTAC |
| Pr11510 | CTCAAGAAAAATCCAAATGGCTACTGC |
| Pr11511 | TCTCCCTTCTTAAACTGACGATCTTCTACGATTAAGATTG |
| Pr11512 | CAATCTTAATCGTAGAAGATCGTCAGTTTAAGAAGGGAGA |
| Pr11525 | GACAAGGCACCATGGTCAGATAACG |
| Pr11526 | GAGACTCGAGGTCGTCAACGAGTAAAATTGTCTTTCCC |
| Pr11527 | GAGATCTAGAGGGATATAAGATAGAGAAACCGAGAGGACA |
| Pr11528 | GAGATCTAGAGGGATATAAGATAGAGAAACCGAGAGGACA |
| Pr11529 | CAATTTTACTCGTTGACGACAAGATAGAGAAACCGAGAGG |
| Pr11530 | CCTCTCGGTTTCTCTATCTTGTCGTCAACGAGTAAAATTG |
| Pr11761 | CAGATGAGGATGAATTGGTTCGTCAG |
| Pr11762 | GAGATCTAGACTCGAGCTGCATAACATCCGCAAAATCGTC |
| Pr11763 | GAGACTCGAGAAGGGAACCACTTTTGCAGAGAATTTG |
| Pr11764 | GCTTCTGGCTTACGAAAAAGGG |
| Pr11933 | CTAGCAGCATAGATAGCGAGATTTC |
| Pr11934 | GCTCTAGACTCGAGTCTATTAATAAAATCTTGTGCATGCG |
| Pr11935 | GAGACTCGAGGGATTTATCGAGACCAAGAAAGGAA |
| Pr11936 | TATAAATCTCCAGACCACCTTCCTT |
| Pr11941 | AATTCTCCCTTGACTTGTGACAATC |
| Pr11942 | GAGGTACCCTCGAGCAATTTCAGAATGTGTTCTTCATCAT |
| Pr11943 | GAGACTCGAGCAATTTATTCGAACCATTCGGGGTTAT |
| Pr11944 | GAAGTAACTGGTGTCCTCAATTCAT |
| Pr11945 | ATGATATGTTGGATCGATTGGAAAAGA |
| Pr11946 | GCTCTAGACTCGAGCAGACCTTCTGTCACCATGTACTCAT |
| Pr11947 | GAGACTCGAGTGCGGAAAATCGCCAAAAGCCTACC |
| Pr11948 | ATAGTCTTTCAAGCGCGTCACAAAA |
| Pr11949 | GTATCTTGGGTCAATTCATAACCGA |
| Pr11950 | GCTCTAGACTCGAGCTCACGGATCATCTCTTCATCTTCTA |
| Pr11951 | GAGACTCGAGACTGTGCGCAATGTTGGTTATAAATT |
| Pr11952 | CTTCATTCTTTTCATGCAAGTCCG |
| Pr11953 | TGTATGGACATTTCTCCTTGCTCAA |
| Pr11954 | GAGGTACCCTCGAGACACATGGCATCTCGCAACATACTTT |
| Pr11955 | GAGACTCGAG GGTAATCGAACAGAGGCAGCTAATA |
| Pr11956 | AGAGGGTATTGACAACAGAAACTTC |
| Pr11957 | TGGCTAGAAAACCAAGAATAGAGTT |
| Pr11958 | GCTCTAGACTCGAGTTCAATTCTAGTCTGTTGGGAAAAAT |
| Pr11959 | GAGACTCGAG CGTTATAAGGTCAGGGAAGTGTCTG |
| Pr11960 | CCGACTATAGCGTTCCATATCTCTA |
| Pr12040 | AAATCCGTGGTTTTCGACAT |
| Pr12041 | GCTCTAGACTCGAGGACTTTTCCCGTTGTCTTGTATGA |
| Pr12042 | GAGACTCGAGCAAAAGTGATTGACAATTAGCAAGA |
| Pr12043 | CTGATGGAACAGCCATTTGA |
| Pr12044 | AACCCTGCAAACAGAAGTGG |
| Pr12045 | GCTCTAGACTCGAGCATTTCCTCGTCATCTGCAA |
| Pr12046 | GAGACTCGAGATCGTGACTGTGAAAAATGTTGG |
| Pr12047 | CTGGCTAAAGGGGTTTTCAA |
| Pr12048 | CATGGAGCAGGTTTGCTTCT |
| Pr12049 | GCTCTAGACTCGAGGTGCGTGGTGTTGGTTACAC |
| Pr12050 | GAGACTCGAGCCCCATGGCTGACCTACTTA |
| Pr12051 | TGATGCAACACTTGCTAGGC |
| Pr12419 | CGCAGGTGATGGTATGTTGA |
| Pr12422 | TTCGTTGATTTCTGGGTTGA |
| Pr13543 | ATTATCTATTGTCGGTAGGGATGCC |
| Pr13544 | GCTCTAGACTCGAGCAATCACTACAGGTAGGATGGGAA |
| Pr13545 | CCGCTCGAGAATCAGTCAGAAACATCCAACGGAA |
| Pr13546 | CGATCAGGACAGTCAAATCGATTTC |
| Pr13547 | ATCCTTGCGGTAGAAATGCCTGTTAAGACAGGTCT |
| Pr13548 | GGCATTTCTACCGCAAGGATGGCGATATCTACAGACTCCT |
| Pr14944 | GGTGAATAATTGGGGTTAAATTAATT |
| Pr14945 | GCTCTAGACTCGAGTTCCTTGTCTTTTATTTTCTTACTC |
| Pr14946 | GAGACTCGAGAGTTTTATTTGGTTTGACTATTTACCT |
| Pr14947 | TTACATGTATGGACATTTCTCCTTGCT |
| Pr14950 | CTGGGACATCCTTTTGCTATGCTTAGTGTCAAGATAGATCTAGCC |
| Pr14951 | ATAGCAAAAGGATGTCCCAGACTATCATGCAAATCCTGACCGATA |
| Pr15171 | CCATTATATTGAGGAAGTGGAACAC |
| Pr15172 | ATCTGCTTCTCGAGTGACTACTTGT |
| Pr15173 | AGAATTTTTATTGACCAAACGACAA |
| Pr15174 | GTTTTCTGCCAGTTGTTCATCTTAT |
| Pr15175 | CACAAGTGGAAAAGGAATTAAGAGA |
| Pr15176 | ACCAACTCAAGCAAAATCATAGAAG |
| Pr15196 | TCCCAGACTATCGGCCAAATCCTGACCGATACGATTGCGC |
| Pr15197 | GGTCAGGATTTGGCCGATAGTCTGGGACATACCTTTGCTA |
| Pr15211 | AACCAAATAAAACTTTCCTTGTCTTTTATTTTCTTACTCA |
| Pr15212 | TAAAAGACAAGGAAAGTTTTATTTGGTTTGACTATTTACC |
| Pr15511 | ATCCTTGAGGTAGAAATGCCTGTTAAGACAGGTCTTGAAG |
| Pr15512 | GGCATTTCTACCTCAAGGATGGCGATATCTACAGACTCCT |
| Pr15732 | CCATTATCATTATGACATCG |
| Pr15733 | GAGACTCGAGTTTAGATTGTATCGTGACTGTG |
| Pr15734 | ATATTTTGGATGATCTTTGT |
| Pr15735 | GAGATCTAGACTCGAGGGGATATAAGATAGAGAAACCGAGA |
| Pr15736 | GAGACTCGAG CTCGTCGTCAACGAGTAAAATTGTCTTTCCC |
| Pr15737 | ATGGTATGTTGACTCGTCTCTTGCT |
| Pr15738 | GAGATCTAGACTCGAGCAGATTGCAGGTGTGACACCTC |
| Pr15739 | GAGACTCGAG ACCTTGTCTTACCAGATATTCATCT |
| Pr15740 | AAGTCTTTACATGTCTGGAC |
| Pr15741 | GAGACTCGAG ATCTTCTGCGACTAATACTTTCAT |
| Pr15742 | GAGATCTAGACTCGAGGGTAATCGAACAGAGGCAGCTAATA |
| Pr15743 | TCGGCCTCAAGCAGGCAAGTATCTT |
| Pr15862 | GGTGATAATTTTGACTGGGAACA |
| Pr15863 | AAACCCCGATTCATTACCAA |
| Pr15864 | TGTCGATGGTCAGTGGGTTA |
| Pr15865 | TTCCCTTACGTTGCGTATCC |
| Pr15866 | CTCGTAAACCAGACCGCTTC |
| Pr15867 | CATTTGGAACCATCTGAGCA |
| Pr15868 | ACCTTCTTTGCTCACCGTGT |
| Pr15869 | TTGTACAAAGGCACCACCAA |
| Pr15870 | CCTGCTATCATGGGGTCAGT |
| Pr15871 | GCGTCAATCAAAGCTGTCAA |
| Pr15872 | TTGATCATGGAAGGTCGTGA |
| Pr15873 | AGCCCGCTAACAGGTCATTA |
| Pr15874 | GAAAGTGTGGCGACTGTTGA |
| Pr15875 | TGGCAAATCCAGCATCATTA |
| Pr15876 | AAAATGTGGAGGCGATTGAC |
| Pr15877 | CATTCATTGTTTTGGCTGGA |
| Pr15878 | CAGAGGTGGATGCTGACAAA |
| Pr15879 | TCACCCCAGATATTGCCTTC |
| Pr15880 | GGCAAGGACAACTTTGGGTA |
| Pr15881 | TCGTTTGAGCAGGATTAGCC |
| Pr15882 | CGATTGCTGTAGCGATTCAA |
| Pr15883 | GCAACCCAAGGTAAATCCAA |
| Pr15884 | TGCAGGGGAAGTTAAAGCAG |
| Pr15885 | GCTTCCGATAATTCCGATGA |
| Pr15886 | TGATTGTCTTGGCAGTGAGC |
| Pr15887 | TCTGCAAGGAGACCAAGTCA |
| Pr15888 | TGGAGCCAATGTCAAACGTA |
| Pr15889 | AGGAATACCTGGGGCAAAAG |
| Pr15890 | TGAAAATCAAAGAGCAAACTAGGA |
| Pr15891 | ATCTCTTCAAACCACGTCAGC |
| Pr15892 | CATCAATGCCCTTTGGTTCT |
| Pr15893 | TGATAATGCCGACCACGTAA |
| Pr15894 | GGCTATGCCCATCATCATCT |
| Pr15895 | GCAGTCAGCCCACCTTTTAG |
| Pr15896 | GCAGATGCTTGGACACTCAA |
| Pr15897 | CGAAGGATGTCCATGACCTT |
| Pr15898 | GTGCTTCTGGTCATGCTCAA |
| Pr15899 | GTGCTTCTGGTCATGCTCAA |
| Pr15900 | GCCGTATCGCTTAACCACAT |
| Pr15901 | CTAGCTCGTCTCCACCTTCG |
| Pr15902 | AGCCTTTACAACTGCGGCTA |
| Pr15903 | ACCATACGTTGGCTGAATCC |
| Pr15920 | AGTAGGGCGATAAAATCGTTTATCAC |
| Pr15923 | TTTTCAGCAAGGTTACGTTCTAAAC |
| Pr15924 | TGGTAGATATAAACGACACCGTTTG |
| Pr15925 | GAGATCTAGACTCGAGTCGTATCTCAAATCGTGTCATAAAAC |
| Pr15926 | GAGACTCGAGATTCACGAATATCAAAAGTAAGACA |
| Pr15927 | TGGCTTCTGGAGTTTTGGCAGGAAC |
| Pr15928 | CGTTTCACGTCCCAGTTCGCTCATC |
| Pr15929 | GAGATCTAGACTCGAGCCCTACAAGTACAATTTTCATCTTT |
| Pr15930 | GAGACTCGAGAAGAACTGCTTTTTAAGTAGTTCTTT |
| Pr15931 | AGGCTTTATAAACCTCTACATGATAG |
| Pr15934 | GAGACTCGAGATTCTCTATCCTAACTCCCATTTAC |
| Pr15935 | TGAATACGCTTGATAAGTAGCTTC |
| Pr15936 | TCTTTAGCAACTTCTCTTGTTCACA |
| Pr15937 | GAGATCTAGACTCGAGCTGAATTGGATGTGAAGACATGTAT |
| Pr15938 | GAGACTCGAGCTTTATATTCCTAAAGTATAATTTT |
| Pr15939 | TGCAGCTACGACGACTTGACCATTA |
| Pr15946 | GAGATCTAGATCAACAAGAAATAAAACCCCGATTC |
| Pr15947 | GAGACTCGAGGGTAATTTTAGTAAATTTCCGAACT |
| Pr15948 | ACTGTGTCGGACGGTTTTCT |
| Pr15949 | GAGATCTAGACTCGAGGGGGCAGTGAAGTAATACTCAATGA |
| Pr16174 | GTTGCTTCTATTCTTATCCCTCTCC |
| Pr16175 | TTTTTATAACAACCCAATTCATAGGT |
| Pr16178 | GACACCAGAACAACTTAAAGCAAGT |
| Pr16179 | CACTCCCAAGTATCAGGAATATCAT |
