# Supplementary material for: Regulation of pneumococcal epigenetic and colony phases by multiple two-component regulatory systems
Source: PLoS Pathog. 2020 Mar 18;16(3):e1008417. doi: 10.1371/journal.ppat.1008417 (PMC7105139; doi:10.1371/journal.ppat.1008417)
Supplement: S7 Table — (DOCX) [file ppat.1008417.s007.docx]

**Table S7. PCR amplifications used for pneumococcal mutagenesis in this study**

| Strain ID | Genotype | Primers | | | Template DNA | Parental strain |
| --- | --- | --- | --- | --- | --- | --- |
|  |  | Upstream | Downstream | Digestion/Fusion |  |  |
| TH6986 | ∆*rr03*::JC1 | Pr10418/Pr10419 | Pr10420/Pr10421 | XbaI/XhoI | ST606 | ST606 |
| TH7009 | ∆*rr03* | Pr10454/Pr10455 | Pr10456/Pr10457 | XhoI/XhoI | ST606 | TH6986 |
| TH8425 | ∆*rr09*::JC1 | Pr11507/Pr11508 | Pr11509/Pr11510 | XbaI/XhoI | ST606 | ST606 |
| TH8468 | ∆*rr09* | Pr11507/Pr11512 | Pr11511/Pr11510 | Pr11507/Pr11510 | ST606 | TH8425 |
| TH9025 | ∆*rr01*::JC1 | Pr11933/Pr11934 | Pr11935/Pr11936 | XbaI/XhoI | ST606 | ST606 |
| TH9029 | ∆*rr04*::JC1 | Pr11941/Pr11942 | Pr11943/Pr11944 | XbaI/XhoI | ST606 | ST606 |
| TH9031 | ∆*rr07*::JC1 | Pr11945/Pr11946 | Pr11947/Pr11948 | XbaI/XhoI | ST606 | ST606 |
| TH9033 | ∆*rr10*::JC1 | Pr11949/Pr11950 | Pr11951/Pr11952 | XbaI/XhoI | ST606 | ST606 |
| TH9035 | ∆*rr11*::JC1 | Pr11953/Pr11954 | Pr11955/Pr11956 | XbaI/XhoI | ST606 | ST606 |
| TH9037 | ∆*rr13*::JC1 | Pr11957/Pr11958 | Pr11959/Pr11960 | XbaI/XhoI | ST606 | ST606 |
| TH9048 | ∆*rr01* | Pr11933/Pr11934 | Pr11935/Pr11936 | XhoI/XhoI | ST606 | TH9025 |
| TH9054 | ∆*rr04* | Pr11941/Pr11942 | Pr11943/Pr11944 | XhoI/XhoI | ST606 | TH9029 |
| TH9057 | ∆*rr07* | Pr11945/Pr11946 | Pr11947/Pr11948 | XhoI/XhoI | ST606 | TH9031 |
| TH9060 | ∆*rr10* | Pr11949/Pr11950 | Pr11951/Pr11952 | XhoI/XhoI | ST606 | TH9033 |
| TH9063 | ∆*rr11* | Pr11953/Pr11954 | Pr11955/Pr11956 | XhoI/XhoI | ST606 | TH9035 |
| TH9066 | ∆*rr13* | Pr11957/Pr11958 | Pr11959/Pr11960 | XhoI/XhoI | ST606 | TH9037 |
| TH9114 | ∆*rr06*::JC1 | Pr12044/Pr12045 | Pr12046/Pr12047 | XbaI/XhoI | ST606 | ST606 |
| TH9115 | ∆*rr06*::JC1 | Pr12044/Pr12045 | Pr12046/Pr12047 | XbaI/XhoI | ST606 | ST606 |
| TH9116 | ∆*rr12*::JC1 | Pr12040/Pr12041 | Pr12042/Pr12043 | XbaI/XhoI | ST606 | ST606 |
| TH9118 | ∆*rr14*::JC1 | Pr12048/Pr12049 | Pr12050/Pr12051 | XbaI/XhoI | ST606 | ST606 |
| TH9164 | ∆*rr06* | Pr12044/Pr12045 | Pr12046/Pr12047 | XhoI/XhoI | ST606 | TH9114 |
| TH9167 | ∆*rr14* | Pr12048/Pr12049 | Pr12050/Pr12051 | XhoI/XhoI | ST606 | TH9118 |
| TH9180 | ∆*rr08*::JC1 | Pr11527/Pr11528 | Pr11525/Pr11526 | XbaI/XhoI | ST606 | ST606 |
| TH9181 | ∆*rr08* | Pr11525/Pr11530 | Pr11529/Pr11528 | Pr11525/Pr11528 | ST606 | TH9180 |
| TH9259 | ∆*rr12* | Pr12040/Pr12041 | Pr12042/Pr12043 | XhoI/XhoI | ST606 | TH9116 |
| TH9553 | *rr06* rev | Pr12044 | Pr12047 |  | ST606 | TH9115 |
| TH9555 | *rr14* rev | Pr12048 | Pr12051 |  | ST606 | TH9118 |
| TH10415 | ∆*rr05*::JC1 | Pr11761/Pr11762 | Pr11763/Pr11764 | XbaI/XhoI | ST606 | ST606 |
| TH10784 | ∆*rr05* | Pr11761/Pr11762 | Pr11763/Pr11764 | XhoI/XhoI | ST606 | TH10415 |
| TH10790 | *rr05* rev | Pr11761 | Pr11764 |  | ST606 | TH10415 |
| TH10793 | *rr08* rev | Pr12419 | Pr12422 |  | ST606 | TH9180 |
| TH10796 | *rr09* rev | Pr11507 | Pr11511 |  | ST606 | TH8425 |
| TH10799 | *rr11* rev | Pr11953 | Pr11956 |  | ST606 | TH9035 |
| TH11403 | ∆*tcs11*::JC1 | Pr13543/Pr13544 | Pr11955/Pr11956 | XbaI/XhoI | ST606 | ST606 |
| TH11425 | *rr11*^D53A^ | Pr11953/Pr13548 | Pr11956/Pr13547 | Pr11953/Pr11956 | ST606 | TH9035 |
| TH11849 | *tcs11* rev | Pr13543 | Pr11956 |  | ST606 | TH11403 |
| TH11863 | ∆*tcs11* | Pr13543/Pr13544 | Pr11955/Pr11956 | XbaI/XhoI | ST606 | TH11403 |
| TH13068 | *psrA*^Y247A^ ∆*rr06*:JC1 | Pr12044 | Pr12047 |  | TH9114 | TH6552 |
| TH13070 | *psrA*^Y247A^ ∆*rr08*:JC1 | Pr11528 | Pr11525 |  | TH9180 | TH6552 |
| TH13072 | *psrA*^Y247A^ ∆*rr09*:JC1 | Pr11507 | Pr11510 |  | TH8425 | TH6552 |
| TH13074 | *psrA*^Y247A^ ∆*rr14*:JC1 | Pr12048 | Pr12051 |  | TH9118 | TH6552 |
| TH13076 | *psrA*^Y247A^ ∆*rr11*::JC1 | Pr11953 | Pr11956 |  | TH9035 | TH6552 |
| TH13115 | *psrA*^Y247A^ ∆*rr06* | Pr12044 | Pr12047 |  | TH9164 | TH13068 |
| TH13117 | *psrA*^Y247A^ ∆*rr08* | Pr11528 | Pr11525 |  | TH9181 | TH13070 |
| TH13119 | *psrA*^Y247A^ ∆*rr09* | Pr11507 | Pr11510 |  | TH8468 | TH13072 |
| TH13121 | *psrA*^Y247A^ ∆*rr14* | Pr12048 | Pr12051 |  | TH9167 | TH13074 |
| TH13123 | *psrA*^Y247A^ ∆*rr11* | Pr11953 | Pr11956 |  | TH9063 | TH13076 |
| TH13125 | *psrA*^Y247A^ *rr11*^D53A^ | Pr11953 | Pr11956 |  | TH11425 | TH13076 |
| TH13127 | *psrA*^Y247A^ *rr11* rev | Pr11953 | Pr11956 |  | ST606 | TH13076 |
| TH13418 | *∆*MYY1924-1925::JC1 | Pr14944/Pr14945 | Pr14946/Pr14947 | XbaI/XhoI | ST606 | ST606 |
| TH13428 | *∆*MYY1924-1925(*rr11* locus) | Pr14944/Pr15211 | Pr15212/Pr14947 | Pr14944/Pr15176 | ST606 | TH13418 |
| TH13455 | ∆*hk11*::JC1 | Pr13543/Pr13544 | Pr13545/Pr13546 | XbaI/XhoI | ST606 | ST606 |
| TH13473 | ∆*hk11* | Pr13543/Pr13544 | Pr13545/Pr13546 | XbaI/XhoI | ST606 | TH13455 |
| TH13475 | *hk11* rev | Pr13543 | Pr13546 |  | ST606 | TH13455 |
| TH13482 | *hk11*^H184A^ | Pr13543/Pr15196 | Pr15197/Pr13546 | Pr13543/Pr13546 | ST606 | TH13455 |
| TH13483 | *hk11*^T190P^ | Pr13543/Pr14951 | Pr14950/Pr13546 | Pr13543/Pr13546 | ST606 | TH13455 |
| TH13757 | *rr11*^D53E^ | Pr11953/Pr15512 | Pr15511/Pr11956 | Pr11953/Pr11956 | ST606 | TH9035 |
| TH13845 | *psrA*^Y247A^ *rr11*^D53E^ | Pr11953/Pr15512 | Pr15511/Pr11956 | Pr11953/Pr11956 | ST606 | TH13076 |
| TH13971 | ST877 ∆*rr06*::JC1 | Pr15732/Pr12045 | Pr15733/Pr15734 | XbaI/XhoI | TH6675 | TH6675 |
| TH13973 | ST877 ∆*rr08*::JC1 | Pr15735/Pr11528 | Pr15736/Pr15737 | XbaI/XhoI | TH6675 | TH6675 |
| TH13975 | ST877 ∆*rr09*::JC1 | Pr11507/Pr15738 | Pr15739/Pr11510 | XbaI/XhoI | TH6675 | TH6675 |
| TH13977 | ST877 ∆*rr10*::JC1 | Pr15743/Pr11950 | Pr11951/Pr11952 | XbaI/XhoI | TH6675 | TH6675 |
| TH13979 | ST877 ∆*rr11*::JC1 | Pr11953/Pr15741 | Pr15742/Pr11956 | XhoI/XbaI | TH6675 | TH6675 |
| TH13981 | P384 ∆*rr06*::JC1 | Pr15732/Pr12045 | Pr15733/Pr15734 | XbaI/XhoI | TH6671 | TH6671 |
| TH13983 | P384 ∆*rr08*::JC1 | Pr15735/Pr11528 | Pr15736/Pr15737 | XbaI/XhoI | TH6671 | TH6671 |
| TH13985 | P384 ∆*rr09*::JC1 | Pr11507/Pr15738 | Pr15739/Pr11510 | XbaI/XhoI | TH6671 | TH6671 |
| TH13987 | P384 ∆*rr10*::JC1 | Pr15743/Pr11950 | Pr11951/Pr11952 | XbaI/XhoI | TH6671 | TH6671 |
| TH13989 | P384 ∆*rr11*::JC1 | Pr15740/Pr15741 | Pr15742/Pr11956 | XhoI/XbaI | TH6671 | TH6671 |
| TH13998 | ST877 ∆*rr06* | Pr15732/Pr12045 | Pr15733/Pr15734 | XhoI/XhoI | TH6675 | TH13971 |
| TH14000 | ST877 ∆*rr08* | Pr15735/Pr11528 | Pr15736/Pr15737 | XhoI/XhoI | TH6675 | TH13973 |
| TH14002 | ST877 ∆*rr09* | Pr11507/Pr15738 | Pr15739/Pr11510 | XhoI/XhoI | TH6675 | TH13975 |
| TH14004 | ST877 ∆*rr10* | Pr15743/Pr11950 | Pr11951/Pr11952 | XhoI/XhoI | TH6675 | TH13977 |
| TH14006 | ST877 ∆*rr11* | Pr11953/Pr15741 | Pr15742/Pr11956 | XhoI/XhoI | TH6675 | TH13979 |
| TH14008 | P384 ∆*rr06* | Pr15732/Pr12045 | Pr15733/Pr15734 | XhoI/XhoI | TH6671 | TH13981 |
| TH14010 | P384 ∆*rr08* | Pr15735/Pr11528 | Pr15736/Pr15737 | XhoI/XhoI | TH6671 | TH13983 |
| TH14012 | P384 ∆*rr09* | Pr11507/Pr15738 | Pr15739/Pr11510 | XhoI/XhoI | TH6671 | TH13985 |
| TH14014 | P384 ∆*rr10* | Pr15743/Pr11950 | Pr11951/Pr11952 | XhoI/XhoI | TH6671 | TH13987 |
| TH14016 | P384 ∆*rr11* | Pr15740/Pr15741 | Pr15742/Pr11956 | XhoI/XhoI | TH6671 | TH13989 |
| TH14092 | *∆*MYY134-139::JC1 | Pr15924/Pr15925 | Pr15926/Pr15927 | XbaI/XhoI | ST606 | ST606 |
| TH14095 | *∆*MYY2067-2068::JC1 | Pr15936/Pr15937 | Pr15938/Pr15939 | XbaI/XhoI | ST606 | ST606 |
| TH14097 | *∆*MYY403-408::JC1 | Pr15928/Pr15929 | Pr15930/Pr15931 | XbaI/XhoI | ST606 | ST606 |
| TH14105 | *∆*MYY1793-1796::JC1 | Pr15948/Pr15949 | Pr15934/Pr15935 | XbaI/XhoI | ST606 | ST606 |
| TH14106 | *∆*MYY134-139(*bgaC* locus) | Pr15924/Pr15925 | Pr15926/Pr15927 | XhoI/XhoI | ST606 | TH14092 |
| TH14108 | *∆*MYY403-408(*ugl* locus) | Pr15928/Pr15929 | Pr15930/Pr15931 | XhoI/XhoI | ST606 | TH14097 |
| TH14110 | *∆*MYY2067-2068(*arcA* locus) | Pr15936/Pr15937 | Pr15938/Pr15939 | XhoI/XhoI | ST606 | TH14095 |
| TH14114 | *∆comW*::JC1 | Pr15920/Pr15946 | Pr15947/Pr15923 | XbaI/XhoI | ST606 | ST606 |
| TH14132 | *∆comW* | Pr15920/Pr15946 | Pr15947/Pr15923 | XhoI/XhoI | ST606 | TH14114 |
| TH14193 | *∆*MYY1793-1796(*gtfA* locus) | Pr15948/Pr15949 | Pr15934/Pr15935 | XhoI/XhoI | ST606 | TH14105 |
