# Supplementary material for: Regulation of pneumococcal epigenetic and colony phases by multiple two-component regulatory systems
Source: PLoS Pathog. 2020 Mar 18;16(3):e1008417. doi: 10.1371/journal.ppat.1008417 (PMC7105139; doi:10.1371/journal.ppat.1008417)
Supplement: S8 Table — (DOCX) [file ppat.1008417.s008.docx]

**Table S8. The qRT-PCR settings in this study**

| **Target gene** | **Forward primer** | **Reverse primer** | **Size of amplicon (bp)** |
| --- | --- | --- | --- |
| *comW* | Pr15862 | Pr15863 | 152 |
| MYY134 | Pr15864 | Pr15865 | 164 |
| MYY135 | Pr15866 | Pr15867 | 195 |
| MYY136 | Pr15868 | Pr15869 | 150 |
| MYY137 | Pr15870 | Pr15871 | 185 |
| MYY138 | Pr15872 | Pr15873 | 154 |
| MYY139 | Pr15874 | Pr15875 | 163 |
| MYY403 | Pr15876 | Pr15877 | 177 |
| MYY404 | Pr15878 | Pr15879 | 172 |
| MYY405 | Pr15880 | Pr15881 | 186 |
| MYY406 | Pr15882 | Pr15883 | 167 |
| MYY407 | Pr15884 | Pr15885 | 184 |
| MYY408 | Pr15886 | Pr15887 | 192 |
| MYY1791 | Pr15888 | Pr15889 | 151 |
| MYY1792 | Pr15890 | Pr15891 | 130 |
| MYY1793 | Pr15892 | Pr15893 | 161 |
| MYY1794 | Pr15894 | Pr15895 | 177 |
| MYY1795 | Pr15896 | Pr15897 | 161 |
| MYY1796 | Pr15898 | Pr15899 | 153 |
| *hk11* | Pr15175 | Pr15176 | 232 |
| MYY1924 | Pr15173 | Pr15174 | 228 |
| MYY1925 | Pr15171 | Pr15172 | 220 |
| MYY2067 | Pr15900 | Pr15901 | 154 |
| MYY2068 | Pr15902 | Pr15903 | 159 |
| *era* | Pr7932 | Pr7933 | 175 |
| *hsdS*(non-invertible) | Pr16178 | Pr16179 | 267 |
| *hsdS_A1_* | Pr16174 | Pr16175 | 367 |
