# Supplementary figures and images for: Regulation of pneumococcal epigenetic and colony phases by multiple two-component regulatory systems
Source: PLoS Pathog. 2020 Mar 18;16(3):e1008417. doi: 10.1371/journal.ppat.1008417 (PMC7105139; doi:10.1371/journal.ppat.1008417)

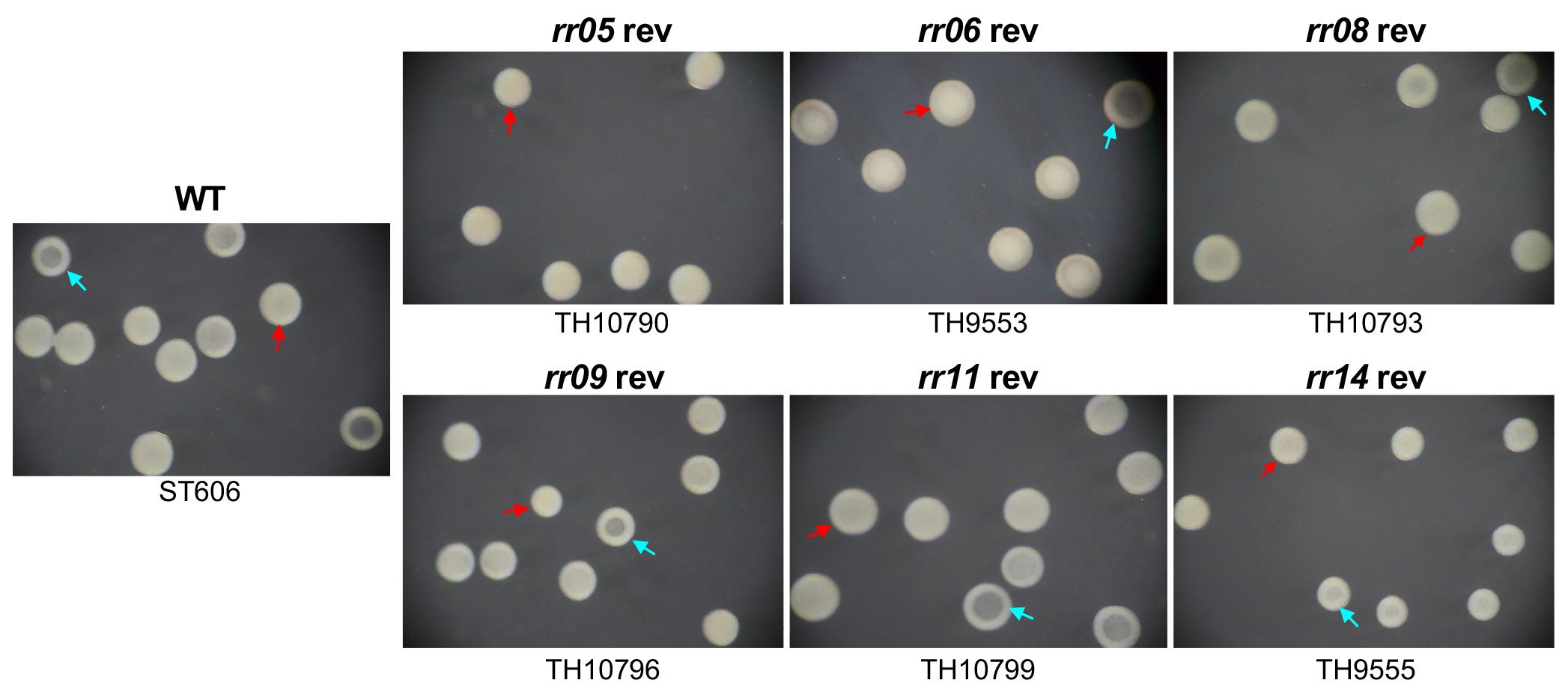

Supplement: S1 Fig — ST606 rr isogenic revertants were grown and processed for photographing of the colonies, and marked as in Fig 1A. (TIF) [file ppat.1008417.s009.tif]

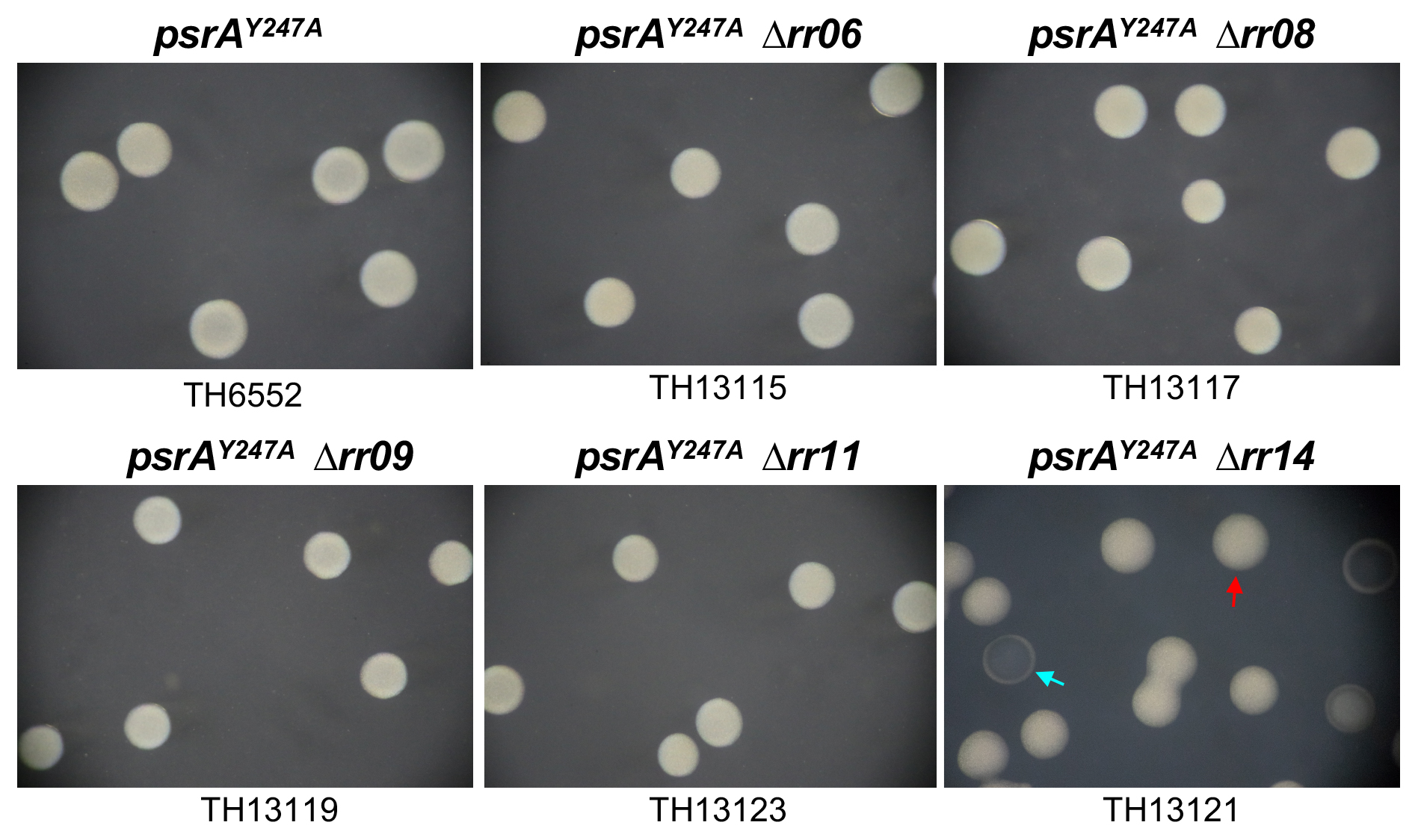

Supplement: S2 Fig — ST606 derivatives with either the inactive psrAY247A allele alone (TH6552) or both the psrAY247A allele and unmarked deletion of a single rr gene were grown and processed for photographing of the colonies, and marked as in Fig 1A. (TIF) [file ppat.1008417.s010.tif]

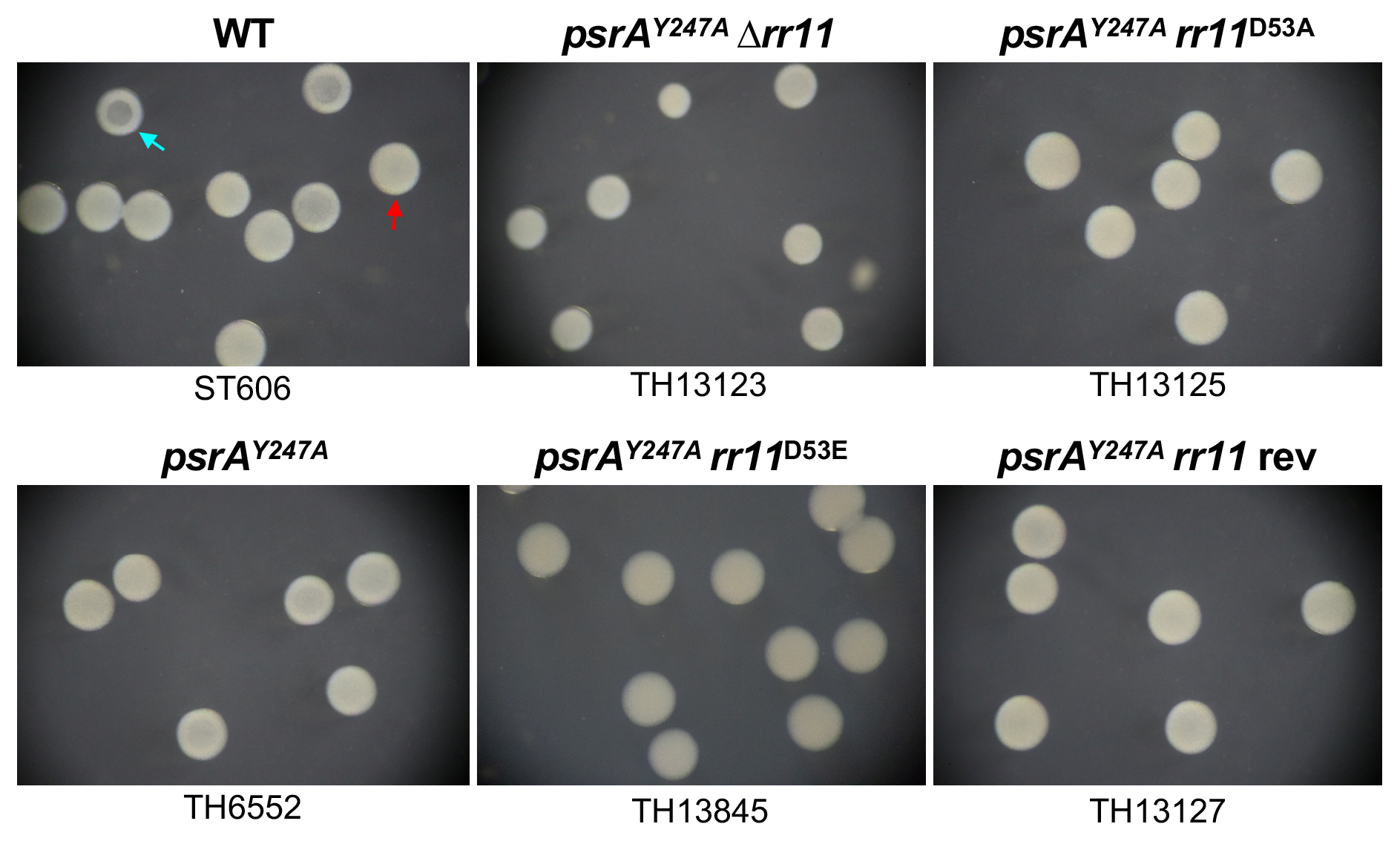

Supplement: S3 Fig — ST606 derivatives with either the inactive psrAY247A allele alone (TH6552) or both the psrAY247A allele and rr11 mutants were grown and processed for photographing of the colonies, and marked as in Fig 1A. (TIF) [file ppat.1008417.s011.tif]

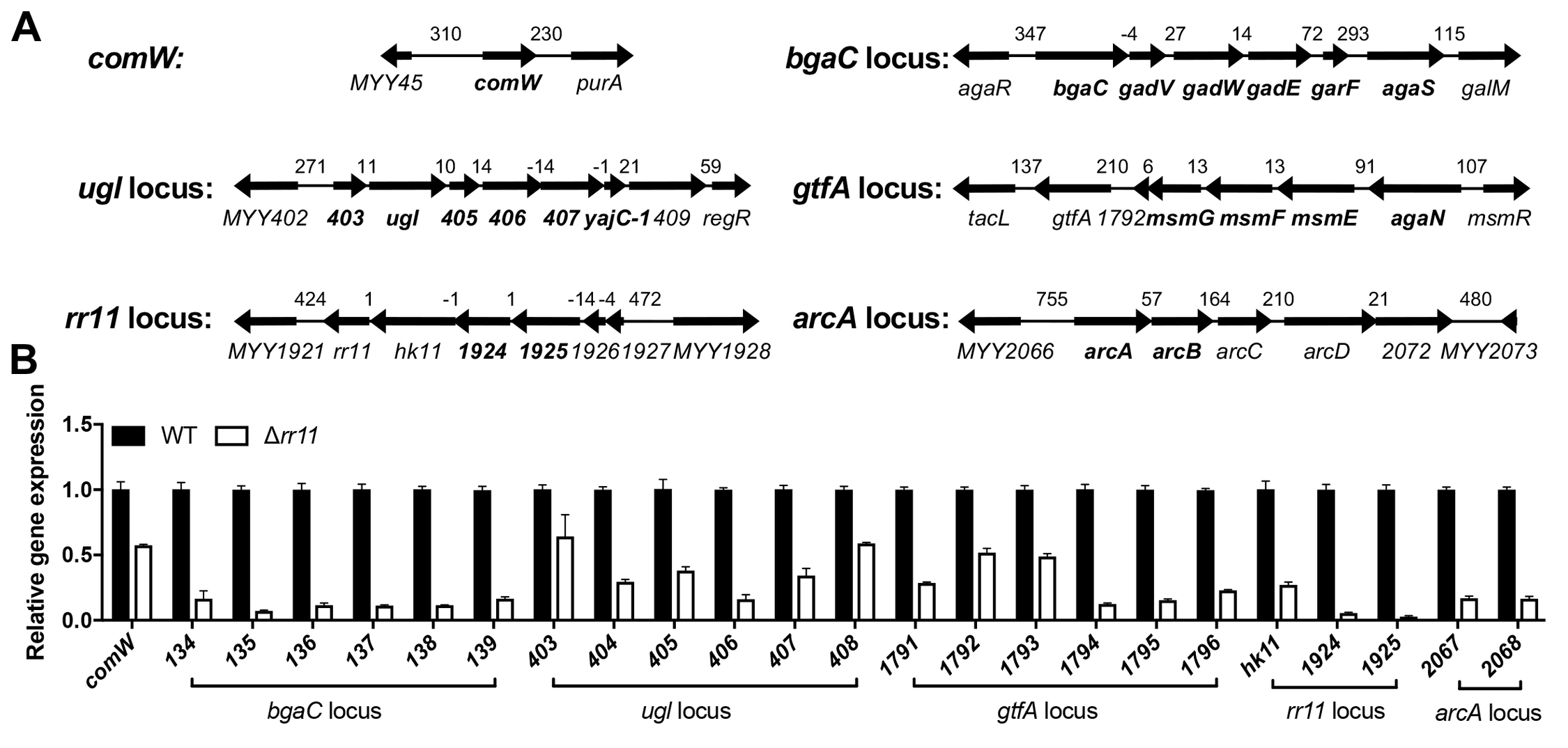

Supplement: S4 Fig — A. The genetic organization of the six RR11-regulated gene loci. The translational orientations of the genes in the six RR11-regulated gene loci are indicated with arrowheads; each gene identified with its functional or genomic names below; the number of nucleotides between two adjacent genes marked at the intergenic region. The genes deleted for mutagenesis were shown in bold. B. Transcription of the RR11-regulated genes in the Δrr11 mutant. Transcriptions of comW and the genes in the loci of MYY134-139, MYY403-408, MYY1791-1796, MYY1923-1925 and MYY2067-2068 in the ST606 (WT) and Δrr11 strains were detected by qRT-PCR. Relative transcriptional difference of each gene in the rr11 mutant is calculated by normalizing the CT value of each gene to that of the parental strain. (TIF) [file ppat.1008417.s012.tif]
